# Systematic pharmacology-based strategy to explore the mechanism of *Semen Strychni* for treatment of papillary thyroid carcinoma

**DOI:** 10.1038/s41598-023-45741-9

**Published:** 2023-10-28

**Authors:** Jingxin Mao, Lijing Tang, Ling Fang, Cheng Tian, Zhaojing Zhu, Yan Li

**Affiliations:** 1 Chongqing Medical and Pharmaceutical College, No. 82, Middle University Town Road, Shapingba District, Chongqing, 400030 China; 2 College of Pharmaceutical Sciences, Southwest University, Chongqing, 400715 China; 3 Chongqing Key Laboratory of High Active Traditional Chinese Drug Delivery System, Chongqing, 400030 China

**Keywords:** Computational biology and bioinformatics, Cancer, Plant sciences

## Abstract

The aim of the study was to investigated the mechanism of *Strychnos nux-vomica* L. (*Semen Strychni, SS*) against papillary carcinoma thyroid (PTC) by combined of network pharmacology and experimental verification. By searching the TCMSP, SEA and SwissTarget Prediction database, the main active ingredients and related targets were obtained. Utilizing Venny 2.1.0 String database and Cytoscape 3.7.2 to screened the intersection target and constructed protein–protein interaction (PPI) network diagram. Using R 4.0.4 software carried out the enrichment analysis of GO and KEGG. HPLC was carried out using LC-20A modular HPLC system to identify the bioactive compound brucine present in *SS*. Molecular docking was performed using Discovery 2019 software. The inhibition rate was detected by CCK8 method. Western blot was used to detect the expression levels of brucine anti-PTC related pathway proteins. 14 active components were screened out, of which 4 main components showed tight relationship with PTC. *SS* may play the anti-PTC role by acting on two main pathways (TNF signaling pathway and MAPK signaling pathway) and mediating various biological functions. HPLC analysis revealed that brucine was a suitable marker for standardization of the *SS*. 4 active components exhibit strong binding energy with core protein. Brucine could significantly reduce the activity of BCPAP cells compared with isobrucine, stigmasterol, (+)-catechin. Brucine may reduce the protein expression levels of IL-6, VEGFA, JUN, TP53, 1L1B, PTGS2, BCL2, CASP3, CASP8, and CASP9 while increase the protein expression levels of BAD, cleaved-CASP3, cleaved-CASP8, and cleaved-CASP9 in BCPAP cells, respectively. The active components of *SS* against PTC mainly include isobrucine, stigmasterol, (+)-catechin, brucine. Among them, brucine exhibits the strongest anti-PTC activity in BCPAP cells, which may reduce the PTC-related protein expression levels. Therefore, *SS* may exhibits the anti-PTC activities through multiple targets and pathways.

## Introduction

Thyroid papillary carcinoma (PTC) is the most common type of differentiated thyroid carcinoma, accounting for 80–85% of the total incidence of thyroid malignant tumors^1,2^. In the past 30 years, the incidence rate of thyroid cancer in most regions of the world has continued to rise, and it has become the fastest growing solid tumor with incidence rate in the world^1,2^. According to the data of the Chinese National Cancer Registration Center (CNCHS), thyroid cancer in China will continue to grow at a rate of 4.5% every year. According to the final data which released by China Cancer Center (CCC) in 2017, the total incidence rate of thyroid cancer was about 10.16/105, ranking seventh in the incidence rate of malignant tumors^3,4^. Among them, PTC is the most common pathological type of thyroid cancer, accounting for more than 85% of thyroid cancer^5^. Most PTC patients have a good prognosis after traditional surgical treatment. Although the prognosis of most PTC patients is relative good, PTC will metastasize to the neck or distant lymph nodes in a few cases. The lymph nodes metastasized by PTC may invade important tissues such as blood vessels, nerves and trachea, resulting in increased difficulty in surgery, which may increase the risk of secondary or multiple operations for PTC patients, and finally affect the prognosis of patients^6,7^.

*Strychnos nux-vomica* L., (*Semen Strychni, SS*), as the mature seed of Yunnan *Strychnos nux* or *Strychnos nux*, is a commonly used Traditional Chinese Medicine (TCM) in clinical practice. It belongs to the liver and spleen meridians, which exhibits the effects of dredging collaterals, relieving pain, dispersing knots and reducing swelling^8^. The chemical components of *SS* are complex and diverse, that mainly including alkaloids, phenolic acids, terpenoids, steroids and glycosides^9–13^. Modern pharmacological studies showed that *SS* has significant pharmacological effects in anti-tumor^14^, anti-rheumatism^15^, analgesia^16^, neuroprotection^17^ and other diseases. *SS* contains a variety of monomers and acts on a variety of cell targets. It is difficult to systematically study the synergistic mechanism of *SS* with conventional methods. Network pharmacology provides a new method for the complex molecular mechanism and the material basis of the efficacy of TCM^18^. Through the analysis of various complex and multi-level interaction networks, the synergistic effects of components and multi-objective drugs and their potential mechanisms are better described. It has been widely used to study the molecular mechanism of TCM, TCM pair and TCM compound on diseases^19^. Therefore, the present study focus on the anti-tumor targets of the active components of *SS* through network pharmacological methods, constructs a systematic visual network diagram for the components target diseases, and analyzes the Gene Ontology (GO) function enrichment and Kyoto Encylopaedia of Genes and Genomes (KEGG) signal pathway of target genes, thereby clarifying the potential molecular mechanism of *SS* on anti-PTC. At the same time, we verified the active substances of *SS* screened in the earlier stage at the cellular and protein levels, providing a basis for *SS* anti-PTC (Supplementary Information).

## Data and methods

### Screening of the active components of *SS* and target prediction

All the chemical components are obtained by using *Strychnos nux-vomica* L. or *Semen Strychni* as the key word, from TCM systematic pharmacology analysis platform (TCMSP, http://tcmspw.com/tcmsp.php). Utilizing the oral bioavailability (OB) ≥ 30% and drug like (DL) ≥ 0.18 as screening conditions, all active components of *SS* were obtained. OB and DL are important indicators for screening active ingredients in different TCM respectively^20^. Using SEA database (http://sea.bkslab.org/) and SwissTarget Prediction databases (http://www.swisstargetprediction.ch/index.php) separately for target prediction of active ingredients of *SS*.

### Acquisition of disease targets

From Genecards database (https://www.genecards.org/), using “PTC”, “papillary thyroid microcarcinoma” and “papillary thyroid cancer” as keywords, search for PTC tumor related target genes, and set target genes with reliable evidence sources (Relevance score > 20). The UniProt database (http://www.uniprot.org/) is used to transform the targets and obtain the gene name of the corresponding targets.

### Acquisition of intersection targets

Using R 4.0.4 software^21^ and Venny 2.1.0 online analysis system (https://bioinfogp.cnb.csic.es/tools/venny) to analysis the intersection target genes of active components related target genes and PTC related target genes, the common action target of disease and drug was obtained. Then using the Metascape database (http://metascape.org/), import the corresponding genes, and obtain Gene ID, the relevant genes of *SS* anti-PTC was finally acquired.

### Construction of “component-target-disease” network diagram

The target genes corresponding to the active ingredients of *SS* and the PTC-related target genes were matched to obtain the common genes. That is, the key targets of *SS* against PTC was obtained. Nodes in the network represent components, targets and diseases respectively; the edge in the network is used to connect drugs and active components, active components and target genes. The whole network showed the relationship among drugs, active components and targets. The “component-target-disease” network diagram was constructed by using Cytoscape 3.7.2 software^22^.

### Construction of protein–protein interaction (PPI) network diagram

Input the intersection target genes obtained into the String online analysis platform (https://cn.string-db.org/), select “Homes sapiens” as the species category, and obtain target interaction PPI network diagram. The results were exported in “TSV” format, and key target genes were screened by using Cytoscape 3.7.2 software.

### GO function enrichment analysis and KEGG pathway enrichment analysis

Using DAVID database (https://david-d.ncifcrf.gov/home.jsp) to carry out the GO function enrichment analysis and KEGG pathway enrichment analysis. After setting the threshold value to *P* < 0.05, take the top 20 GO term and KEGG channels, analyze them with clusterProfiler package of R 4.0.4 software^23^, and draw bubble chart and column chart respectively. In addition, to validated the anti-PTC mechanism of *SS* across the key targets and multiple pathways, the KEGG mapper functional analysis was used to mark the target genes on the pathway associated with PTC.

### Molecular docking verification

The SDF files with 2D structure of monomer compounds from *SS* was retrieved and downloaded respectively in PubChem database (https://pubchem.ncbi.nlm.nih.gov/). Then the PDB file of core target protein structure was downloaded from RCSB database (https://www.rcsb.org/). The Discovery Studio 2019 software was used to verify the docking result between the screened compound small molecules and the core target protein macromolecule with the highest degree.

### Data extraction and patient prognosis analysis of GEPIA database

(Gene Expression Profiling and Integrative Analysis) GEPIA database is an online tool for analyzing tumor and normal samples. Its data source is The Cancer Genome Atlas (TCGA) database (http://portal.gdc.cancer.gov/) and Genotype-Tissue Expression (GTEx) database (http://gtexportal.org/home/). It provides customizable functions, such as tumor/normal differential expression analysis, clinical parameter analysis and survival analysis of tumor patients. Using the Gene Expression Profiling Interactive Analysis 2 (GEPIA2) database (http://gepia.cancer-pku.cn/) to further verify the expression results of IL-6, VEGFA, JUN, TP53, 1L1B and PTGS2. To analyze the differential expression of above genes in different stages of thyroid cancer and its relationship with survival and prognosis. Set the filter conditions in the “Stage Plot” module of expression DIY: (1) Gene: IL-6, VEGFA, JUN, TP53, 1L1B and PTGS2; (2) Use major stage: yes; (3) Data sets selection (cancer name): THCA; (4) Log Scale: yes. Set the filter conditions in the “survival plots” module: (1) Gene: IL-6, VEGFA, JUN, TP53, 1L1B and PTGS2; (2) Methods: Overall Survival (OS); (3) Group cutoff: median; (4) Hazards ratio (HR): yes; (5) 95% confidence interval: yes; (6) Axis units: months; (7) Data sets selection: THCA.

### Experimental verification

#### Materials and instruments

Human thyroid papillary carcinoma cell BCPAP was purchased from the Shanghai Cell Bank of the Chinese Academy of Sciences. *SS* was purchased from Chongqing Traditional Chinese Medicine distribution market at June 2020. Isobrucine (CAS No. 129724-78-3), stigmasterol (CAS No. 83-48-7), (+)-catechin (CAS No. 154-23-4), brucine (CAS No. 5892-11-5) were purchased from Selleck China company respectively. 1640 or DMEM culture medium, DMSO, fetal bovine serum, penicillin streptomycin double antibody, 0.25% trypsin, cell counting kit 8 (CCK-8), and DMSO purchased from Sangon Biotech (Shanghai) Co., Ltd.. RIPA lysis buffer, rabbit derived IL-6 antibody, JUN antibody, TP53 antibody, PTGS2 antibody, VEGFA antibody, 1L1B antibody, BAD antibody, BCL2 antibody, CASP3 antibody, CASP8 antibody, CASP9 antibody, cleaved CASP3 antibody, cleaved CASP8 antibody, cleaved CASP9 antibody, rabbit derived GAPDH/(beta-actin) antibody, HRP goat anti-mouse IgG(H + L) antibody and goat anti-rabbit IgG were purchased from Beyotime Biotechnology Co., Ltd., Nanjing Jiancheng Bioengineering Institute, Santa Cruz Biotechnology, and ThermoFisher Scientific Co., Ltd. respectively. The instrument includes BioRad full-automatic microplate reader, BioRad chemiluminescence gel imaging system, OLYMPUS IX51 inverted microscope, MCO-15AC SANYO CO_2_ constant temperature incubator, Eppendorf 5702R low-speed centrifuge, Shimadzu LC-20A high performance liquid chromatography.

#### HPLC analysis of *SS* and brucine

*SS* were air-dried under laboratory conditions and then ground into small pieces. About 50 g of *SS* was soaked in ethanol solvent (200 mL) for 7 days and filtered with cotton plugs. The ethanol is evaporated under pressure, and the extract is concentrated using a rotary evaporator. Shimadzu LC-20A high performance liquid chromatography (HPLC) with dual solvent pump high pressure gradient system, SPD-20A photodiode array detector and automatic sampler are used for one-dimensional separation. Accurately weigh the total 95% ethanol extract of *SS* and brucine standard respectively, and dissolve them in methanol to make the final concentration 1 mg/mL. Using 0.5 μM organic membrane filtration and preparation for HPLC analysis. The gradient of binary mobile phase composed of ethanol solvent was used for chromatographic elution. The initial gradient condition is set as 5% ethanol, the flow rate is 1.0 mL/min, and the gradient increases to 100% ethanol within 60 min. The column temperature was maintained at 37 °C during the whole process. Shimadzu C18 column (250 mm × 4.6 mm, 5 μm), the detection wavelength is 254 nm. The volume of the total 95% ethanol extract of *SS* and brucine standard injection is 20 μL.

#### Cell culture

BCPAP cell was cultured in 1640 medium containing 10% fetal bovine serum and 1% penicillin streptomycin at 37 °C and 5% CO_2_ incubator. Cell growth was observed under inverted microscope, and cells in logarithmic growth phase were taken for subsequent experiments.

#### CCK8 assay kit

Grouping: different concentration of drug group: 2, 4, 8, 16, 32, 64, 128 µmol/L, 4 active compounds (isobrucine, stigmasterol, (+)-catechin, brucine) of *SS* respectively. Control group: the same volume of DMSO (0.01%). BCPAP cells were treated with 5 × 10^3^ cells (100 μL suspension) was inoculated into 96 well plates. Place it in a 37 °C, 5% CO_2_ incubator for 24 h, and then the cell monolayer will cover the bottom of the hole. The concentration of 4 active compounds added to the cells at 2, 4, 8, 16, 32, 64, 128 µmol/L in complete medium 100 μL, each with 3 double wells. Add 90 μL serum free DMEM and 10 μL CCK8 solution per hole, continue to culture for 2 h, and then measure the absorbance of each hole with the microplate reader at 450 nm wavelength. Repeat the experiment for three times. Cells were continued to cultured for 48 and 72 h, and measured the cell activity in each group at 24, 48, 72 h respectively. Cell viability was measured by CCK8 according to the manufacturer’s instruction^24^. Measured the OD value of each well on the computer for the sample to be tested. The cell survival rate is expressed as a percentage, and calculate the half inhibition rate (IC_50_).

#### Western blot assay kit

Protein blotting is a gold standard method used to identify and quantify specific proteins in complex mixtures extracted from cell or tissue lysates^25^. Western blot method was used to detect the expression levels of anti-PTC related proteins. Brucine with the final concentration of 16, 32, 64, 128 µmol/L was added to the culture dish of BCPAP cells, culture for 48 h, collect cells on ice, extract the total protein of each group of cells by RIPA lysis buffer assay kit. Measure the protein concentration by BCA method, gradually carry out sample loading, electrophoresis, membrane transfer, membrane washing and sealing. Add the corresponding primary antibody (1L1B, IL6, JUN, PTGS2, VEGFA, TP53, BAD, BCL2, CASP3, CASP8, CASP9, cleaved CASP3, cleaved CASP8, cleaved CASP9) in proportion of 1:1000 respectively, incubation at room temperature for 1 h according to instructions, overnight at 4 °C, add the secondary antibody (IgG-HRP-conjugated antibodies) after membrane washing, incubate in a shaking table for 2 h, and wash the membrane with TBST for 3 times, 10 min each time. ECL imaging system is used for luminous development. The experiment is repeated at least 3 times, and the gray value is measured and statistically analyzed.

### Statistical methods

The data obtained were expressed in x ± s, analyzed by SPSS 23.0 statistical software, compared between groups by one-way ANOVA, and the images were drawn by GraphPad Prism 9.0 software. Image J software was used for protein gray scale calculation, and *P* < 0.05 was considered as statistically significant difference.

### Patient and public involvement

No patient involved.

## Results

### Screening of main active ingredients

**Figure 1:**
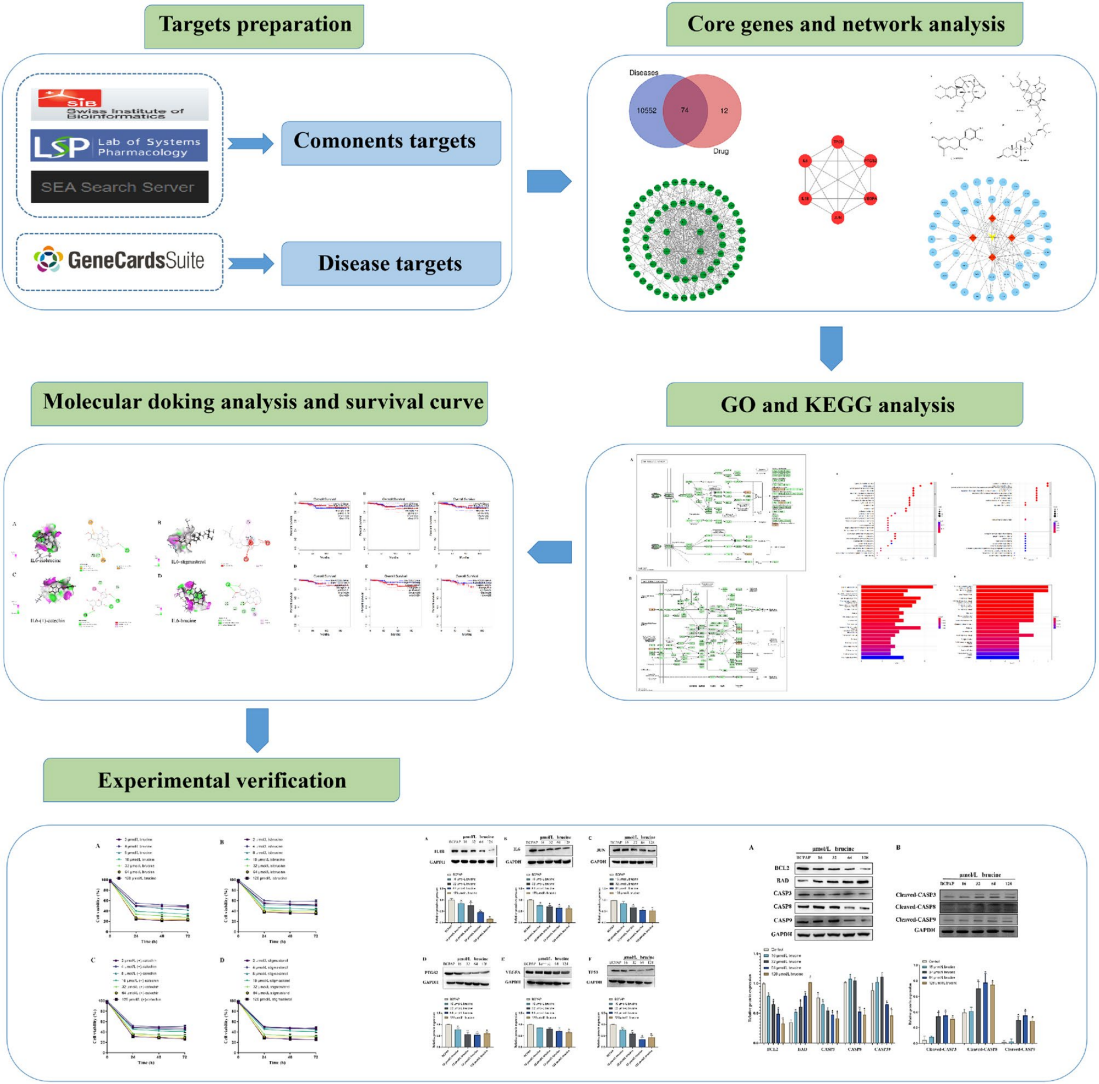
Flow chart of the systematic pharmacology-based strategy study.

The flow chart of the study is shown in Fig. 1. In the present study, 62 chemical components were retrieved through TCMSP database, SEA database and SwissTarget Prediction database. Based on the complexity of data and sample size, DL ≥ 0.18 and OB ≥ 30% were selected as screening conditions. After removing the components that did not find action targets and duplicates, 14 active compounds were finally obtained. The analysis of active ingredient data shows that (+)-catechin, brucine nitrogen oxides have high OB values among these active ingredients, while brucine and strychnine, although their OB and DL values are low, are reported as high content and effective active ingredients in the comprehensive literature, so they are included together. Finally, 14 candidate active compounds of *SS* are determined (Table 1).

**Table 1:** General information of active ingredients of *SS*.

Mol ID	Molecule name	MW	OB (%)	DL
MOL001040	(2R)-5,7-Dihydroxy-2-(4-hydroxyphenyl)chroman-4-one	272.27	42.36	0.21
MOL001476	(S)-Stylopine	323.37	51.15	0.85
MOL003410	Ziziphin_qt	472.78	66.95	0.62
MOL003411	Icaride A	404.5	48.74	0.43
MOL003413	Isostrychnine N-oxide (I)	352.47	35.45	0.8
MOL003414	Isostrychnine N-oxide (II)	350.45	37.33	0.8
MOL003418	Lokundjoside_qt	406.57	32.82	0.76
MOL003432	Vomicine	408.54	47.56	0.65
MOL003429	Strychnine	364.48	7.98	0.48
MOL003440	Brucine N-oxide	410.51	52.63	0.38
MOL003436	Isobrucine	334.45	33.58	0.8
MOL000449	Stigmasterol	412.77	43.83	0.76
MOL000492	(+)-Catechin	290.29	54.83	0.24
MOL003435	Brucine	394.51	7.61	0.41

### Screening of common targets

**Figure 2:**
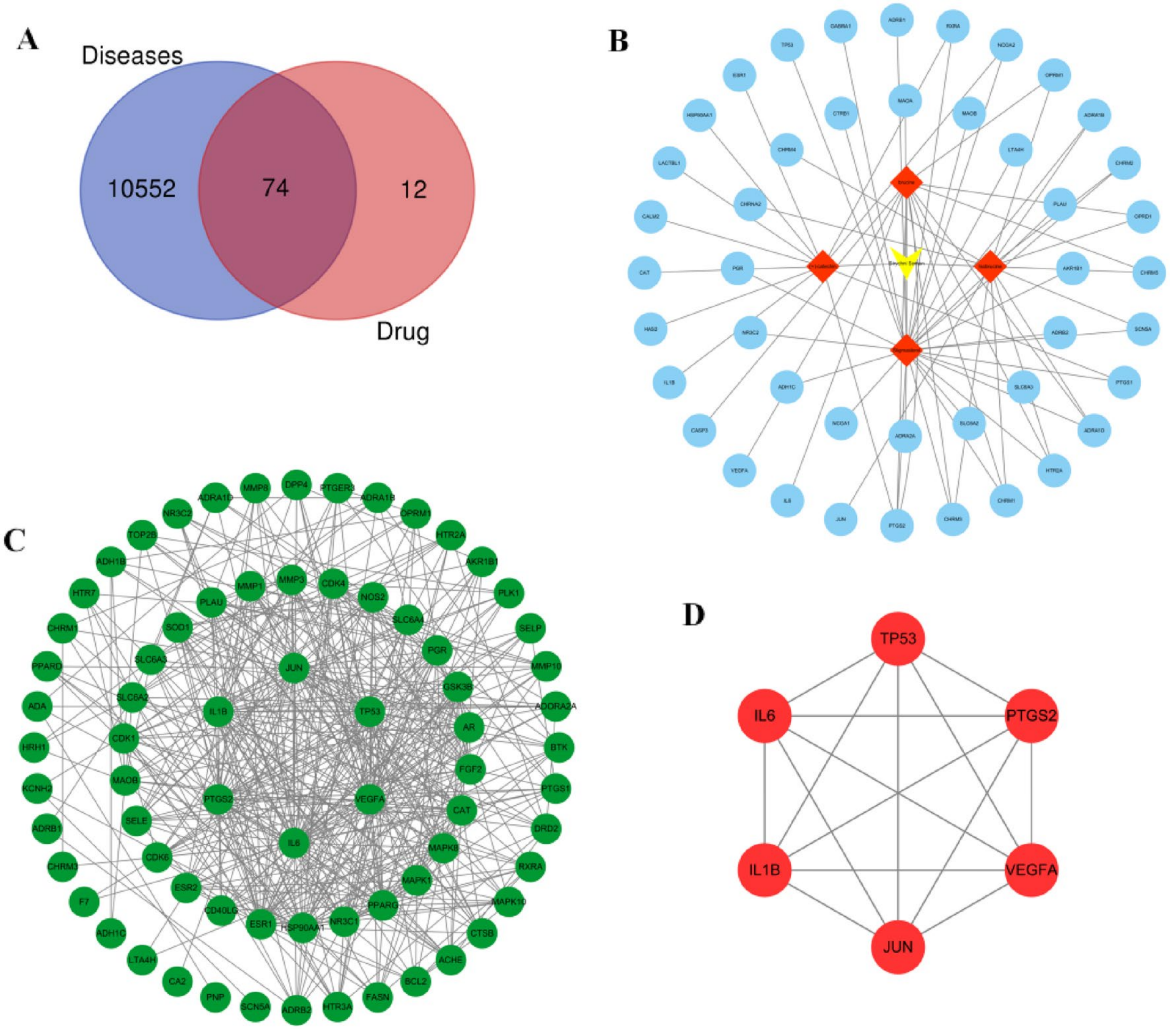
(**A**) Venn diagram of the common target gene screening of *SS* and PTC-related targets. (**B**) Active ingredients of *SS* anti-PTC target network. (**C**) PPI network of *SS* on anti-PTC. (**D**) Key PPI network of *SS* on anti-PTC.

10,626 disease related targets were retrieved from Genecards database, and 86 components related targets of *SS* were screened from TCMSP database, SEA database and SwissTarget Prediction database respectively. Venn diagram shows that there are 74 common targets between diseases and drugs (Fig. 2A). Common targets were screened by R4.0.4 software and Cytoscape 3.7.2 software respectively. According to the highest degree, the first 6 targets were IL6, VEGFA, JUN, TP53, 1L1B and PTGS2 (Table 2).

**Table 2:** Corresponding core targets genes of 4 main ingredients based on the degree value*.*

Gene name	Degree	Betweenness centrality	Closeness centrality
IL6	47	0.20950835	0.72277228
VEGFA	37	0.04372111	0.62931034
TP53	36	0.0446597	0.62931034
JUN	36	0.07763126	0.62931034
IL1B	35	0.05742738	0.64035088
PTGS2	33	0.03094135	0.60330579
ESR1	31	0.05010559	0.60330579
HSP90AA1	29	0.06177295	0.59836066
MMP2	25	0.0090776	0.54887218
CAT	24	0.05559021	0.57480315
NR3C1	24	0.06912668	0.584
PPARG	24	0.01869106	0.57480315
MAPK8	23	0.01237528	0.56589147
MAPK1	22	0.00848556	0.54887218
FGF2	21	0.00427026	0.51048951
AR	20	0.00811866	0.53284672
GSK3B	19	0.00423987	0.53284672
SLC6A4	18	0.05219787	0.5530303
PGR	18	0.01146345	0.53284672
CDK4	16	0.00231747	0.5
NOS2	16	0.02796792	0.52517986
MMP3	15	9.60E−04	0.48666667
MMP1	15	9.54E−04	0.48666667
PLAU	15	0.00131314	0.48666667
SLC6A3	14	0.02029447	0.48993289
MAOA	14	0.02064035	0.46202532
MAOB	13	0.01627505	0.45061728
SLC6A2	13	0.0168167	0.40782123
SOD1	13	0.00235357	0.50694444
SELE	13	0.00121666	0.48993289
CDK6	12	0.00117485	0.48344371
CDK1	12	0.00309293	0.4591195
ACHE	11	0.01378584	0.49324324
DRD2	11	0.00350875	0.43195266
HTR3A	11	0.00406563	0.43452381
CTSB	11	0.00209855	0.48344371
CD40LG	11	0.00241767	0.47712418
ESR2	11	6.15E−04	0.48026316
ADRB2	10	0.0327522	0.49659864
FASN	10	0.00215831	0.48344371

### Construction of drug composition target disease network diagram

With TCMSP and Genecards database as platforms, the relationship between drug components and common targets, and the relationship between common targets and diseases are made into Excel tables, and input into Cytoscape 3.7.2 to generate relevant network diagrams. Since there are 4 components closely related to common targets between common targets and drugs, redundant drug components are deleted and a drug component target disease relationship network diagram is established. In this network diagram, red nodes represent chemical components, while blue nodes plays an anti-PTC role through multi components and multi targets (Fig. 2B).

### Construction of target gene PPI network

Through the String online analysis platform, the interaction data of 74 targets are obtained, and the analysis is carried out using the Cytoscape 3.7.2 software to obtain the PPI network (Fig. 2C). Using R 4.0.4 software and Cytoscape 3.7.2 software to calculate the frequency of target interaction respectively, which are IL6, VEGFA, JUN, TP53, 1L1B and PTGS2 in turn. These targets are the most critical 6 genes in the PPI network of *SS* anti-PTC related targets, which further explains that *SS* acts on these 6 targets, and the targets are interrelated, interacted and synergetic, playing a role in inhibiting tumor cell growth (Fig. 2D).

### GO and KEGG enrichment analysis

**Figure 3:**
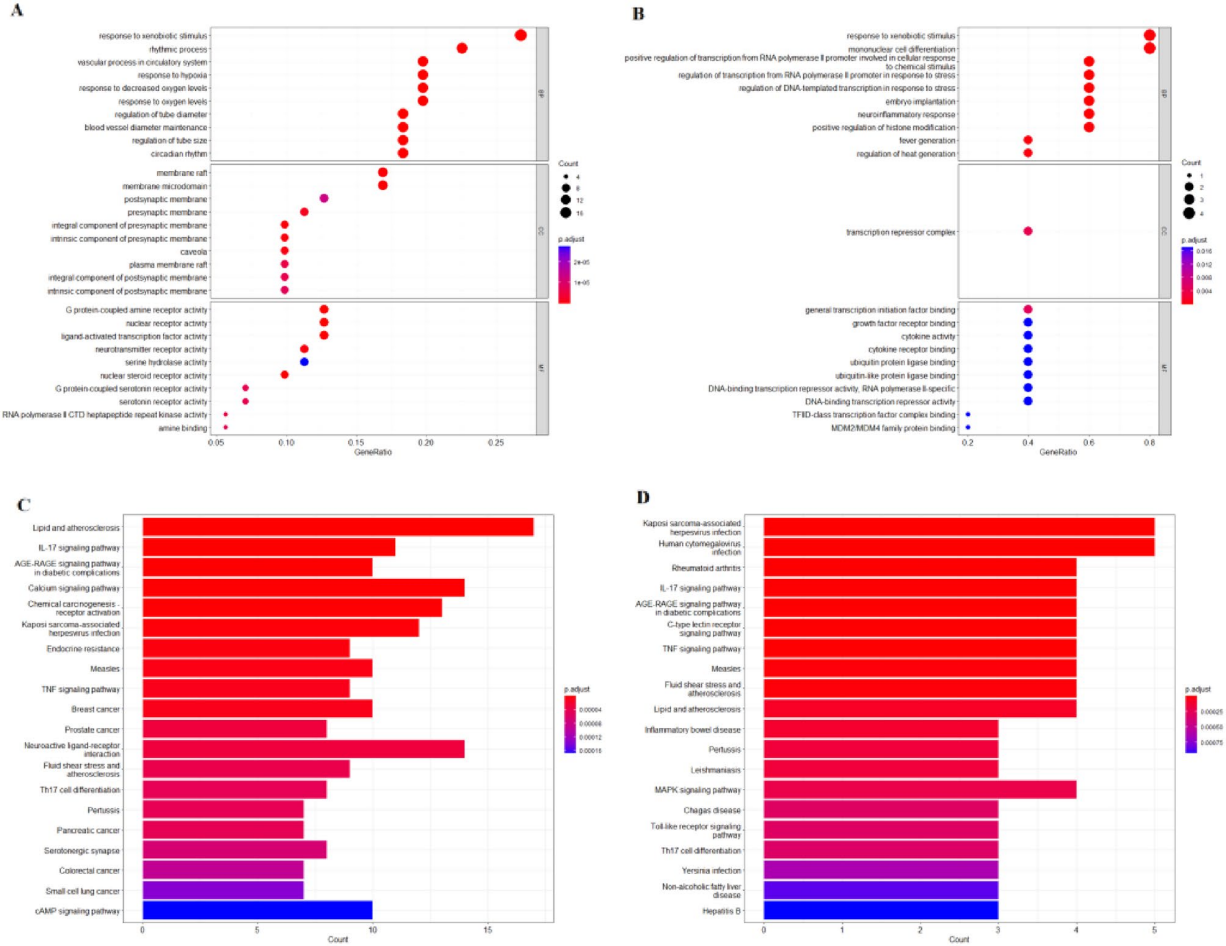
(**A**,**B**) *SS* prevention and treatment of PTC gene GO enrichment analysis bubble diagram. (**C**,**D**) KEGG pathway enrichment histogram chart of the active ingredients of *SS* treatment of PTC.

Through the enrichment analysis of GO on the anti-PTC target of *SS* by R 4.0.4 software, 105 biological processes with* P* < 0.05 were screened, and the first 20 important functional analysis data were visualized. It can be concluded that the biological processes involved in the anti-tumor metastasis targets of *SS* by GO biological process enrichment analysis mainly include response to xenobiotic stimulus, rhythmic process, vascular process in circulatory system, response to hypoxia, response to decreased oxygen levels, response to oxygen levels regulation of tube diameter blood vessel diameter maintenance, regulation of tube size, circadian rhythm. membrane raft, membrane microdomain, postsynaptic membrane, presynaptic membrane, integral component of presynaptic membrane, intrinsic component of presynaptic membrane, caveola, plasma membrane raft, integral component of postsynaptic membrane, intrinsic component of postsynaptic membrane. G protein-coupled amine receptor activity, nuclear receptor activity, ligand-activated transcription factor activity, neurotransmitter receptor activity, serine hydrolase activity, nuclear steroid receptor activity, G protein-coupled serotonin receptor activity, serotonin receptor activity, RNA polymerase II CTD heptapeptide repeat kinase activity, amine binding and so on (Fig. 3A). The core targets mainly involve biological processes including response to xenobiotic stimulus, mononuclear cell differentiation, positive regulation of transcription from RNA polymerase II promoter involved in cellular response to chemical stimulus, regulation of transcription from RNA polymerase II promoter in response to stress, DNA-templated transcription in response to stress, embryo implantation, neuroinflammatory response, positive regulation of histone modification, fever generation, regulation of heat generation. transcription repressor complex, general transcription initiation factor binding, growth factor receptor binding, cytokine activity, cytokine receptor binding, ubiquitin protein ligase binding ubiquitin-like protein ligase binding, DNA-binding transcription repressor activity, RNA polymerase II-specific, DNA-binding transcription repressor activity TFIID-class transcription factor complex binding, MDM2/MDM4 family protein binding (Fig. 3B). It is suggested that the active components of *SS* may inhibit the formation of PTC by interfering with the above biological processes.

**Figure 4:**
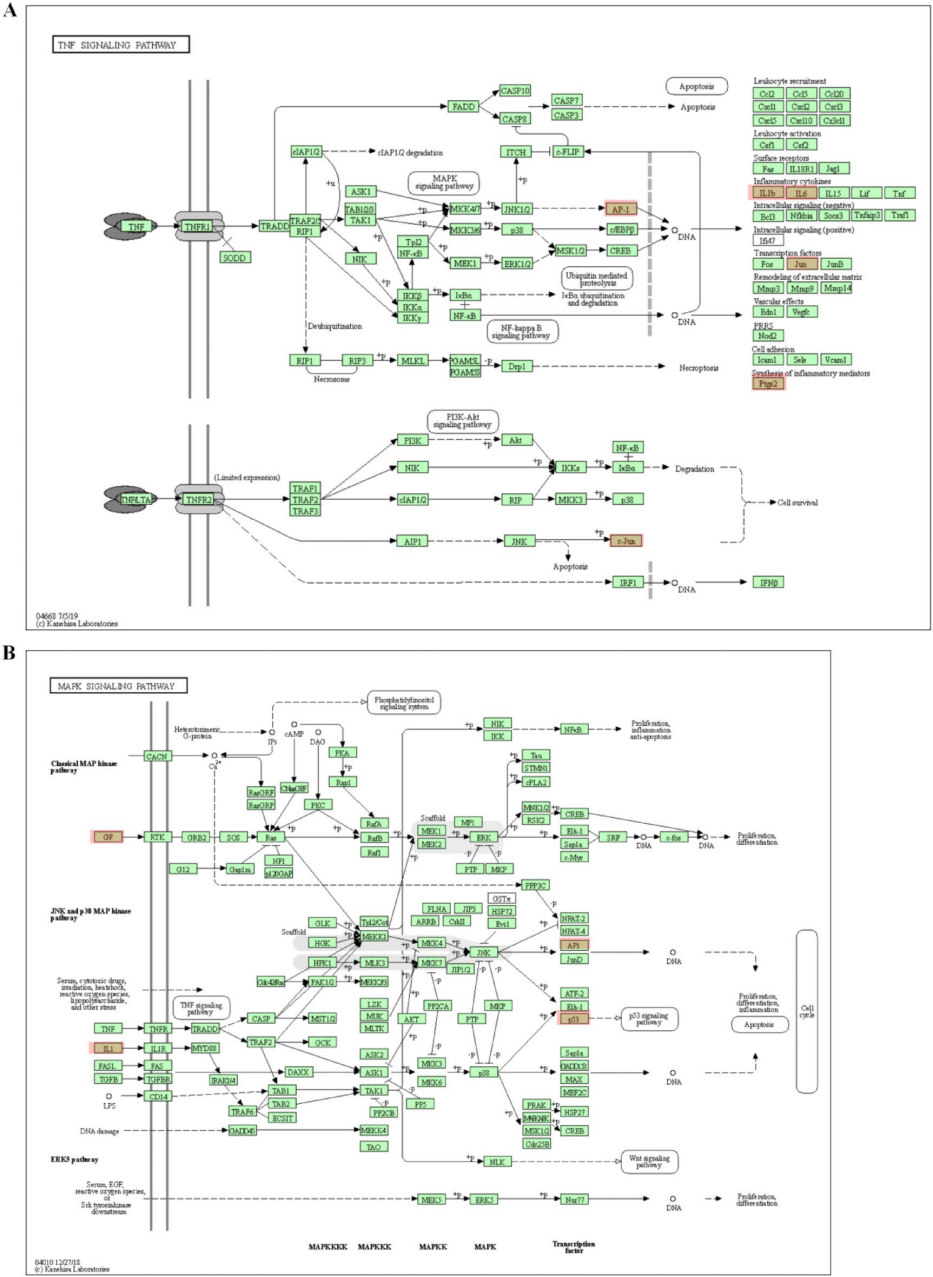
Annotated map of the target genes related the main active components of *SS* on PTC-related signaling pathways. (**A**) TNF signaling pathway. (**B**) MAPK signaling pathway.

Through enrichment analysis of KEGG signal pathway of *SS* target, 117 signal pathways with *P* < 0.05 were screened, and 20 important pathways were visualized by R 4.0.4 software. The pathways involved in the anti-PTC target of *SS* mainly include lipid and atherosclerosis, IL-17 signaling pathway, AGE-RAGE signaling pathway in diabetic complications, calcium signaling pathway, chemical carcinogenesis- receptor activation, kaposi sarcoma-associated herpesvirus infection, endocrine resistance, measles, TNF signaling pathway, breast cancer, prostate cancer, neuroactive ligand-receptor interaction, fluid shear stress and atherosclerosis, Th17 cell differentiation, pertussis, pancreatic cancer, serotonergic synapse, colorectal cancer, small cell lung cancer, cAMP signaling pathway (Fig. 3C). The main anti-PTC targets involve pathways mainly including kaposi sarcoma-associated herpesvirus infection, human cytomegalovirus infection, rheumatoid arthritis, IL-17 signaling pathway, AGE-RAGE signaling pathway in diabetic complications, C-type lectin receptor signaling pathway, TNF signaling pathway, measles, fluid shear stress and atherosclerosis, lipid and atherosclerosis, inflammatory bowel disease, pertussis, leishmaniasis, MAPK signaling pathway, chagas disease, toll-like receptor signaling pathway, Th17 cell differentiation, yersinia infection, non-alcoholic fatty liver disease, Hepatitis B (Fig. 3D). These pathways play an anti-PTC role by regulating the process of tumor generation. In addition, annotated map of the key target genes locations of *SS* in anti-PTC related pathways was presented in Fig. 4. It was found that most of the key target genes are closely with TNF signaling pathway (Fig. 4A) and MAPK signaling pathway (Fig. 4B) in anti-PTC activities respectively.

### Molecular docking results

**Figure 5:**
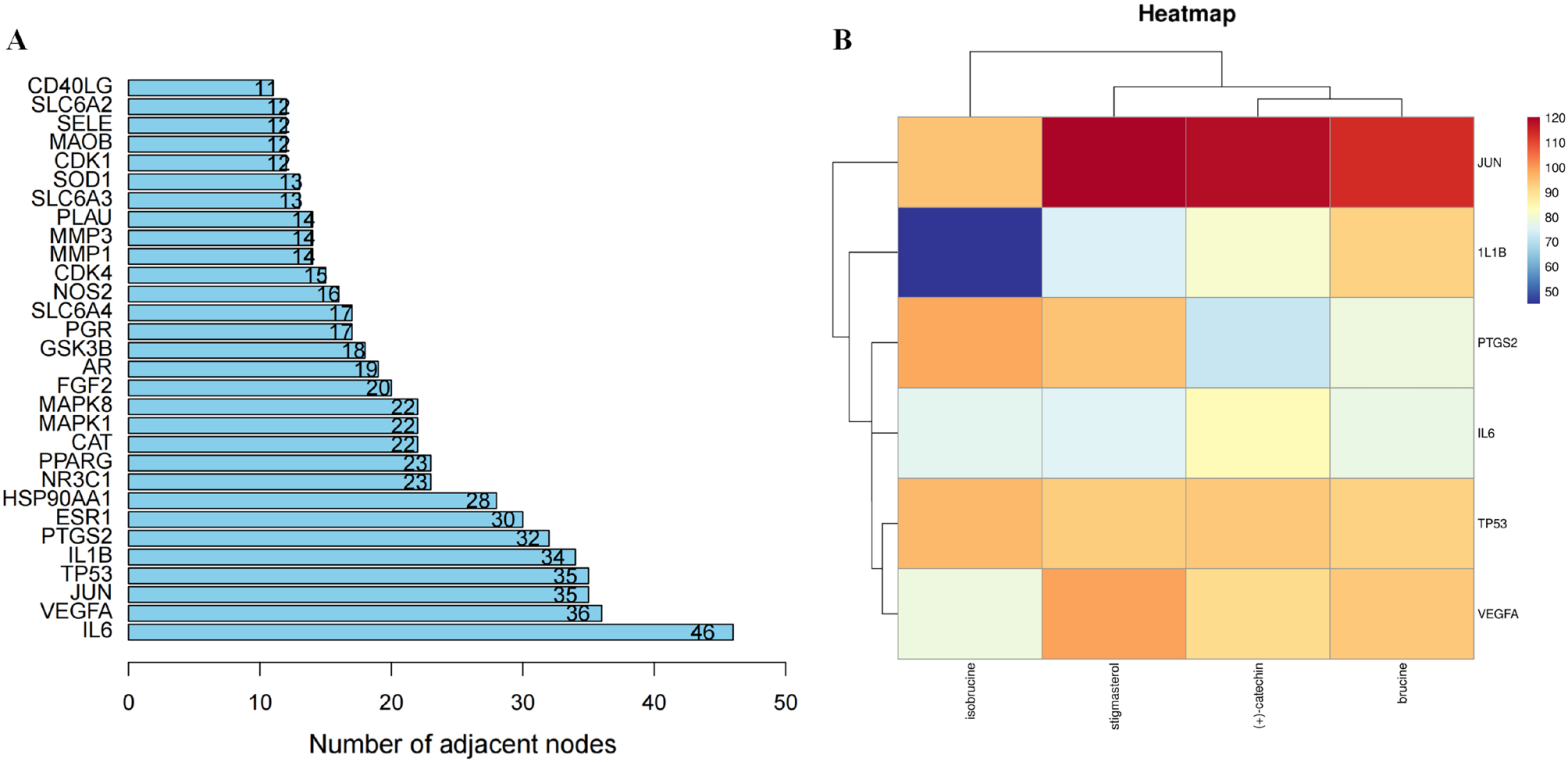
(**A**) Bar diagram of corresponding core targets genes of 4 main ingredients based on the degree value. (**B**) Heatmap of main active ingredients of *SS* with 6 core target genes.

**Figure 6:**
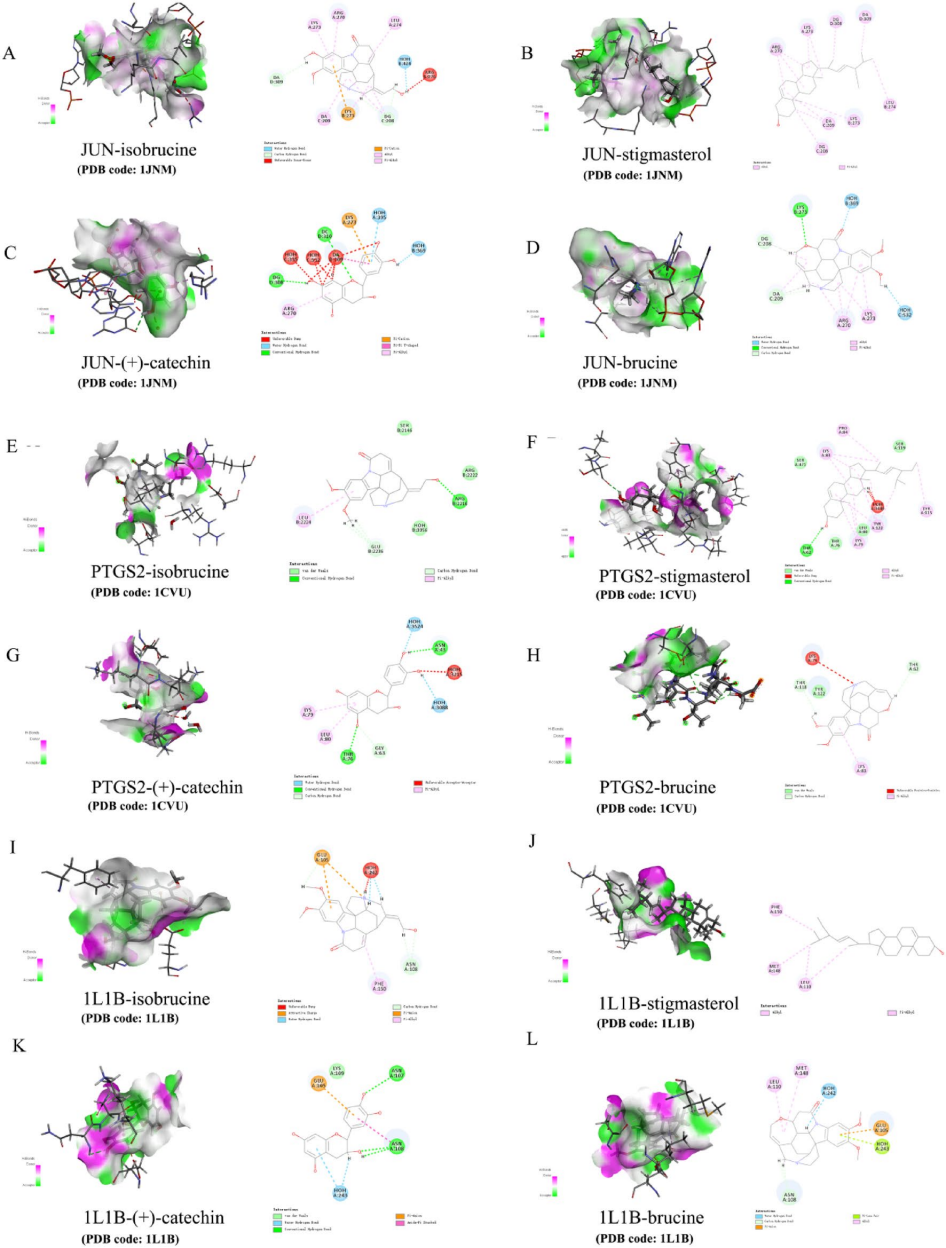
Molecular docking of core target gene JUN, PTGS2, and 1L1B with 4 main ingredients of *SS* respectively. (**A**) isobrucine-JUN, (**B**) stigmasterol-JUN, (**C**) (+)-catechin-JUN, (**D**) brucine-JUN, (**E**) isobrucine-PTGS2, (**F**) stigmasterol-PTGS2, (**G**) (+)-catechin-PTGS2, (**H**) brucine-PTGS2, (**I**) isobrucine-1L1B, (**J**) stigmasterol-1L1B, (**K**) (+)-catechin-1L1B, (**L**) brucine-1L1B.

**Figure 7:**
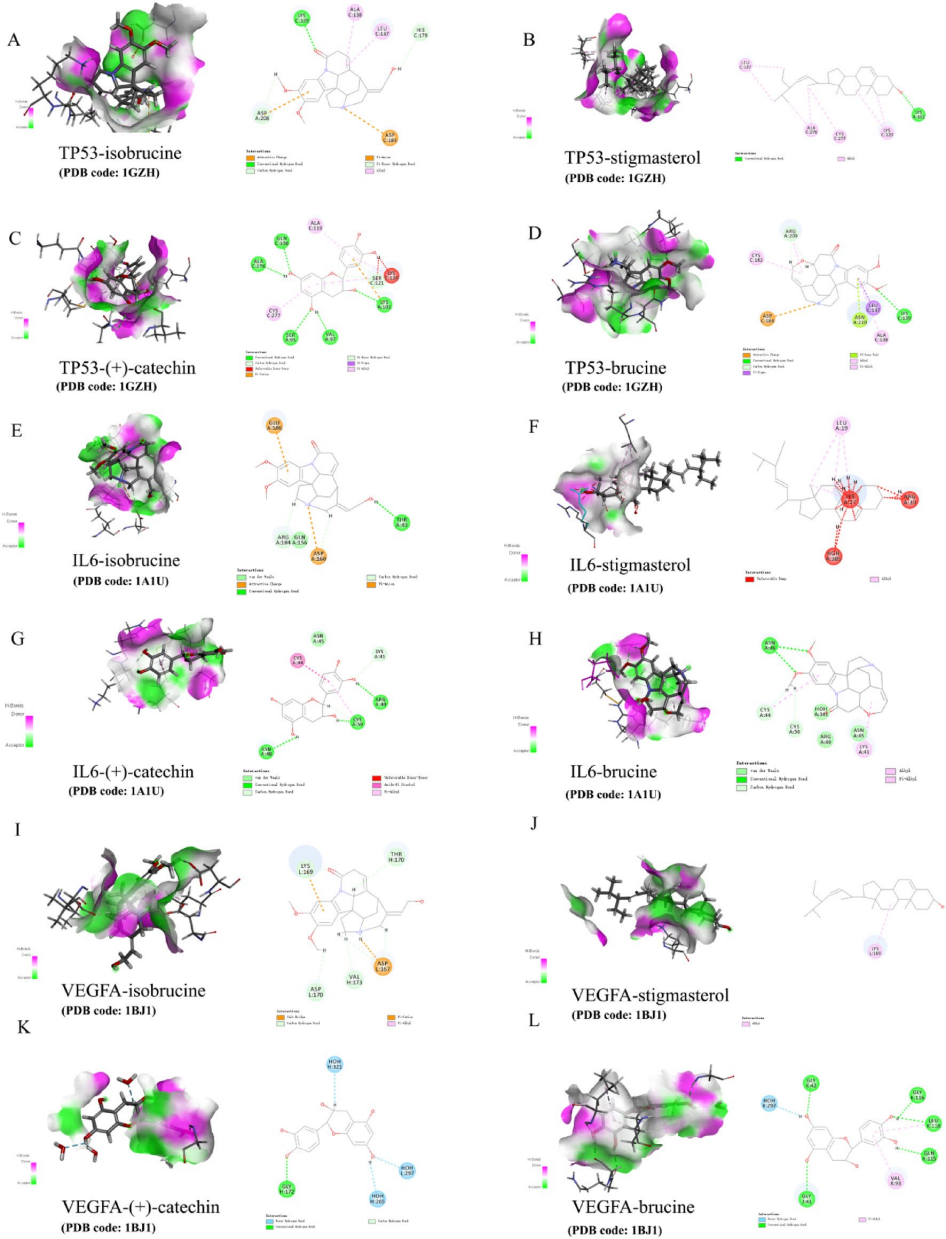
Molecular docking of core target gene TP53, IL6, and VEGFA with 4 main ingredients of *SS* respectively. (**A**) isobrucine-TP53, (**B**) stigmasterol-TP53, (**C**) (+)-catechin-TP53, (**D**) brucine-TP53, (**E**) isobrucine-IL6, (**F**) stigmasterol-IL6, (**G**) (+)-catechin-IL6, (**H**) brucine-IL6, (**I**) isobrucine-VEGFA, (**J**) stigmasterol-VEGFA, (**K**) (+)-catechin-VEGFA, (**L**) brucine-VEGFA.

The structure (SDF format) of each compound retrieved and downloaded from PubChem database was verified by molecular docking with the first 6 selected core targets, and the Discovery Studio 2019 software was used for analysis. The libdock scores was showed in Table 3. The libdock scores were higher, suggesting that it is more likely to be the key drug active molecule of *SS* in the treatment of PTC. The degree of targets (Fig. 5A) and the heatmap of 6 core targets (Fig. 5B) were presented. The active compounds are bound to the target mainly through traditional hydrogen bond, hydrocarbon bond, Pi–Pi conjugation, and base lone pair electron. Among them, stigmasterol (120.188) and (+)-catechin (118.438) have the strongest binding capacity with JUN (Fig. 6A–D), isobrucine (98.8636) and stigmasterol (94.8413) have the strongest binding capacity with PTGS2 (Fig. 6E–H), brucine (92.7554) and (+)-catechin (80.9937) have the strongest binding capacity with 1L1B (Fig. 6I–L), isobrucine (96.5837) and (+)-catechin (93.9245) have the strongest binding capacity with TP53 (Fig. 7A–D), stigmasterol (99.6812) and brucine (94.2969) had the strongest binding ability with VEGFA (Fig. 7E–H), and (+)-catechin (83.85) and brucine (78.1123) had the strongest binding ability with 1L6 (Fig. 7I–L).

**Table 3:** The results of molecular docking.

Compound	Target	PDB	Libdock score
Isobrucine	PTGS2	1CVU	98.8636
Isobrucine	JUN	1JNM	94.6501
Isobrucine	1L1B	1L1B	45.0814
Isobrucine	TP53	1GZH	96.5837
Isobrucine	VEGFA	1BJ1	78.9212
Isobrucine	IL6	1ALU	76.32
Stigmasterol	PTGS2	1CVU	94.8413
Stigmasterol	JUN	1JNM	120.188
Stigmasterol	1L1B	1L1B	75.3339
Stigmasterol	TP53	1GZH	93.1243
Stigmasterol	VEGFA	1BJ1	99.6812
Stigmasterol	IL6	1ALU	75.46
(+)-Catechin	PTGS2	1CVU	71.8213
(+)-Catechin	JUN	1JNM	118.438
(+)-Catechin	1L1B	1L1B	80.9937
(+)-Catechin	TP53	1GZH	93.9245
(+)-Catechin	VEGFA	1BJ1	91.2969
(+)-Catechin	IL6	1ALU	83.85
Brucine	PTGS2	1CVU	78.9863
Brucine	JUN	1JNM	113.797
Brucine	1L1B	1L1B	92.7554
Brucine	TP53	1GZH	92.145
Brucine	VEGFA	1BJ1	94.2969
Brucine	IL6	1ALU	78.1123

### Correlation between the expression of IL-6, VEGFA, JUN, TP53, 1L1B, PTGS2 and the prognosis of PTC

**Figure 8:**
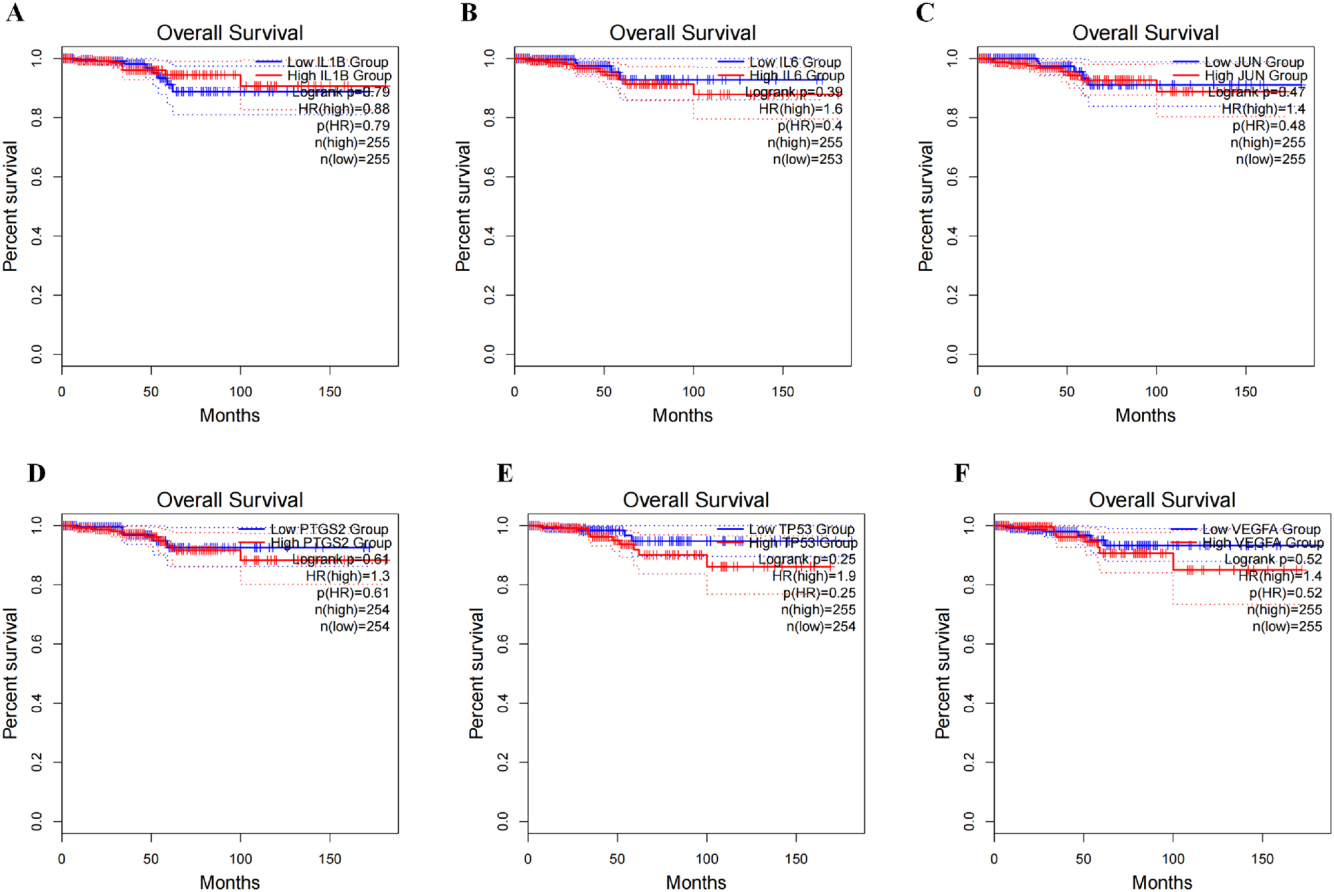
Correlation between the expression of (**A**) 1L1B, (**B**) IL-6, (**C**) JUN, (**D**) PTGS2, (**E**) TP53, (**F**) VEGFA and the prognosis of PTC.

Online survival analysis showed that the expressions of IL-6 (HR = 1.6), VEGFA (HR = 1.4), JUN (HR = 1.4), TP53 (HR = 1.9) and PTGS2 (HR = 1.3) were significantly correlated with the overall survival of PTC respectively. There was no significant correlation between the expression of 1L1B (HR = 0.88) and overall survival (Fig. 8).

### Experimental verification results

#### The results of HPLC

**Figure 9:**
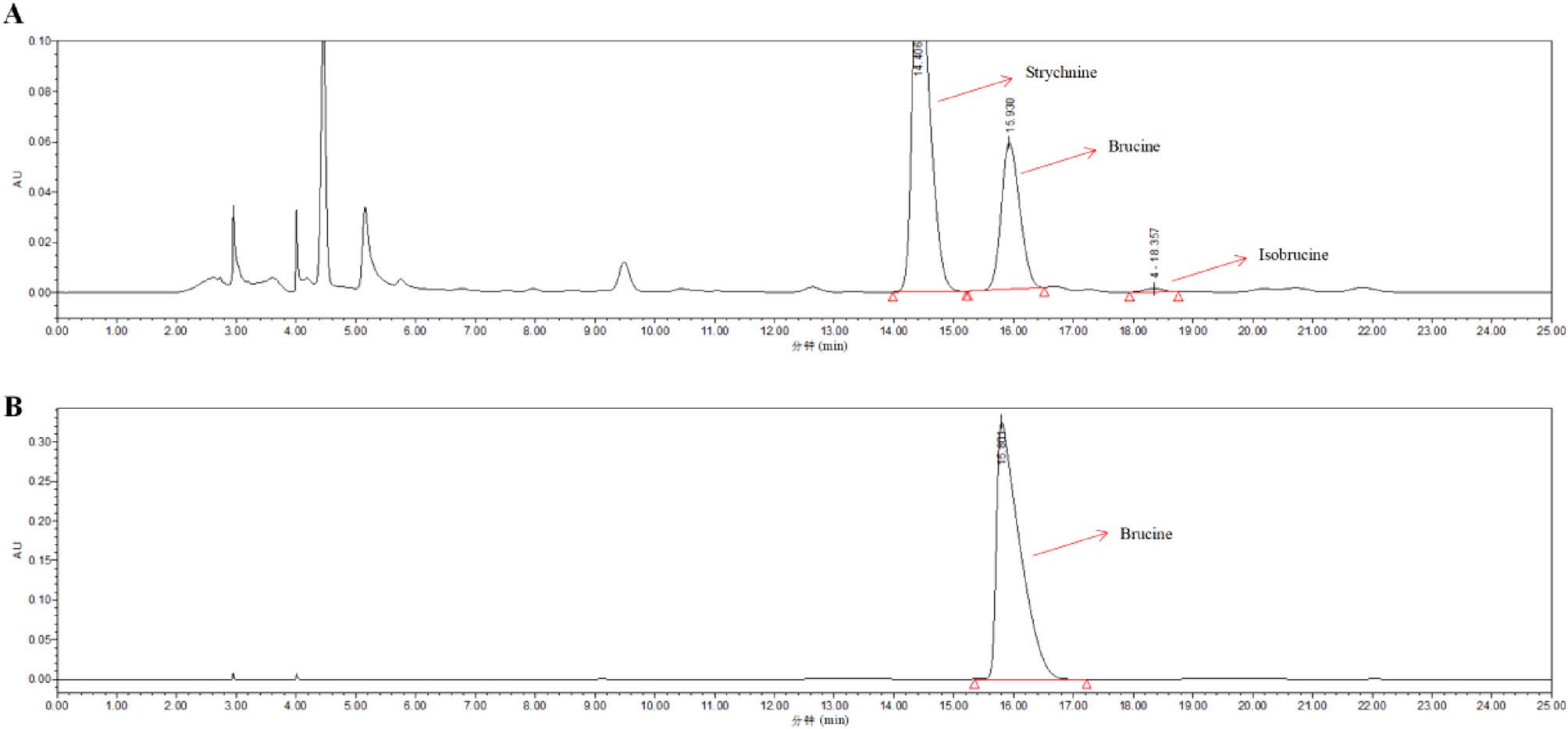
HPLC analysis of (**A**) *SS* ethanol extract and (**B**) brucine respectively.

HPLC analysis of methanol extracts of *SS* was carried out along with brucine standard (Fig. 9). The analysis revealed that brucine standard showed characteristic peak corresponding to *SS*. The retention time for *SS* was 15.930 (Fig. 9A) and for brucine standard was 15.801 (Fig. 9B) respectively. The results showed that brucine was a suitable marker for standardization of the *SS.*

#### The IC_50_ values of active compounds in *SS*

**Figure 10:**
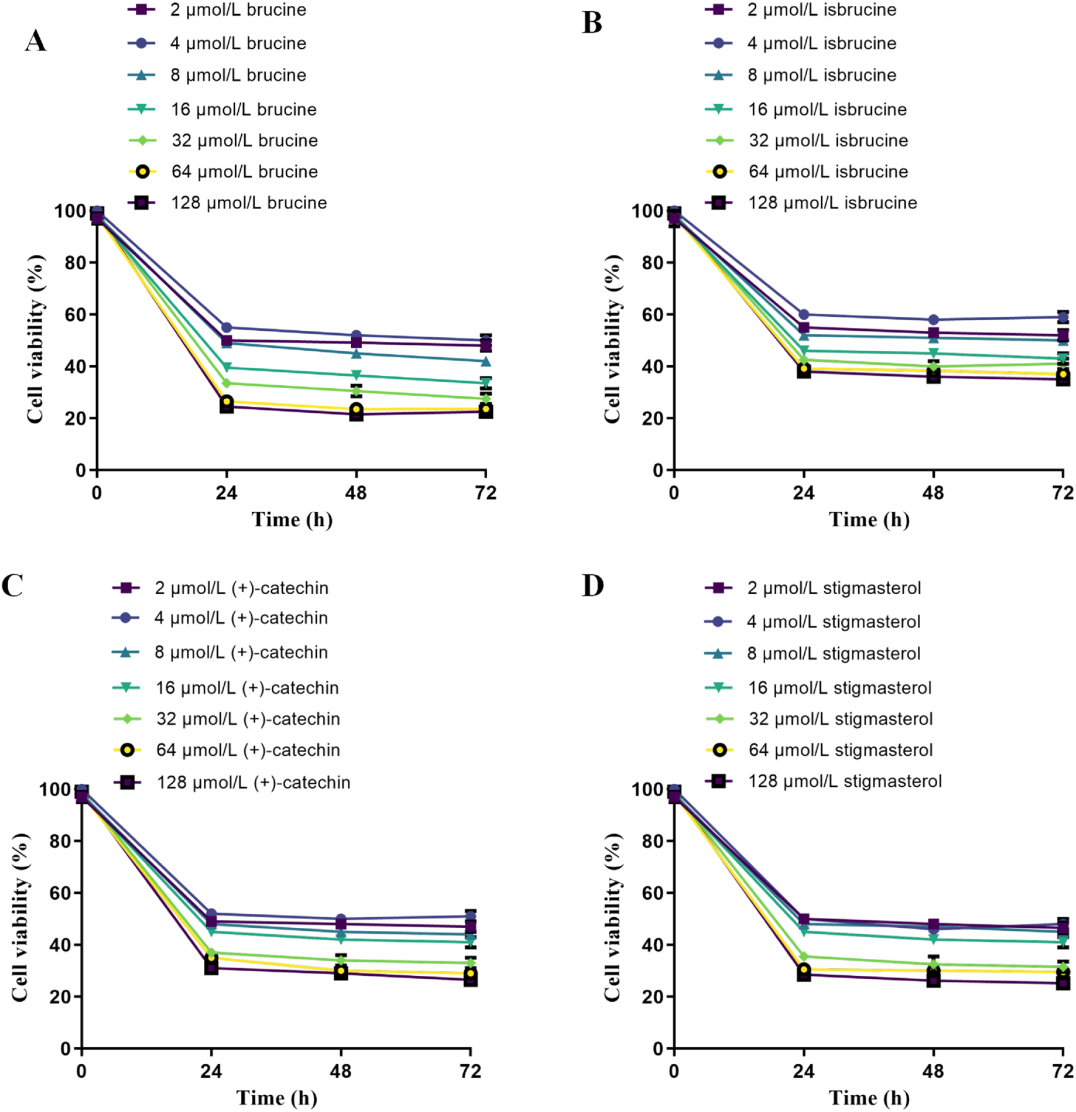
The cell viability of 4 main ingredients of *SS* on BCPAP cell. (**A**) isobrucine, (**B**) stigmasterol, (**C**) (+)-catechin, (**D**) brucine.

The active compounds brucine, isobrucine, (+)-catechin and stigmasterol from *SS* with different concentrations showed certain inhibition rates on BCPAP cells after 24, 48 and 72 h of treatment, respectively. The IC_50_ values of brucine at 24, 48 and 72 h were 0.313, 0.265, 0.201 µmol/mL respectively (Fig. 10A). The IC_50_ values of isobrucine at 24, 48 and 72 h were 0.435, 0.375 and 0.309 µmol/mL respectively (Fig. 10B). The IC_50_ values of (+)-catechin at 24, 48 and 72 h were 0.448, 0.377 and 0.351 µmol/mL respectively (Fig. 10C). The IC_50_ values of stigmasterol at 24, 48 and 72 h were 0.366, 0.315, 0.302 µmol/mL respectively (Fig. 10D). Among them, brucine showed the strongest inhibition on the growth of BCPAP cells (IC_50_ value was the lowest). Therefore, brucine was selected as the most effective compound of *SS* in subsequent experiments. It was revealed that brucine has a significant dose effect relationship on the inhibition of BCPAP cells but without time effect relationship, and the inhibition rate is the highest.

#### Effect of brucine on the expression of PTC related proteins

**Figure 11:**
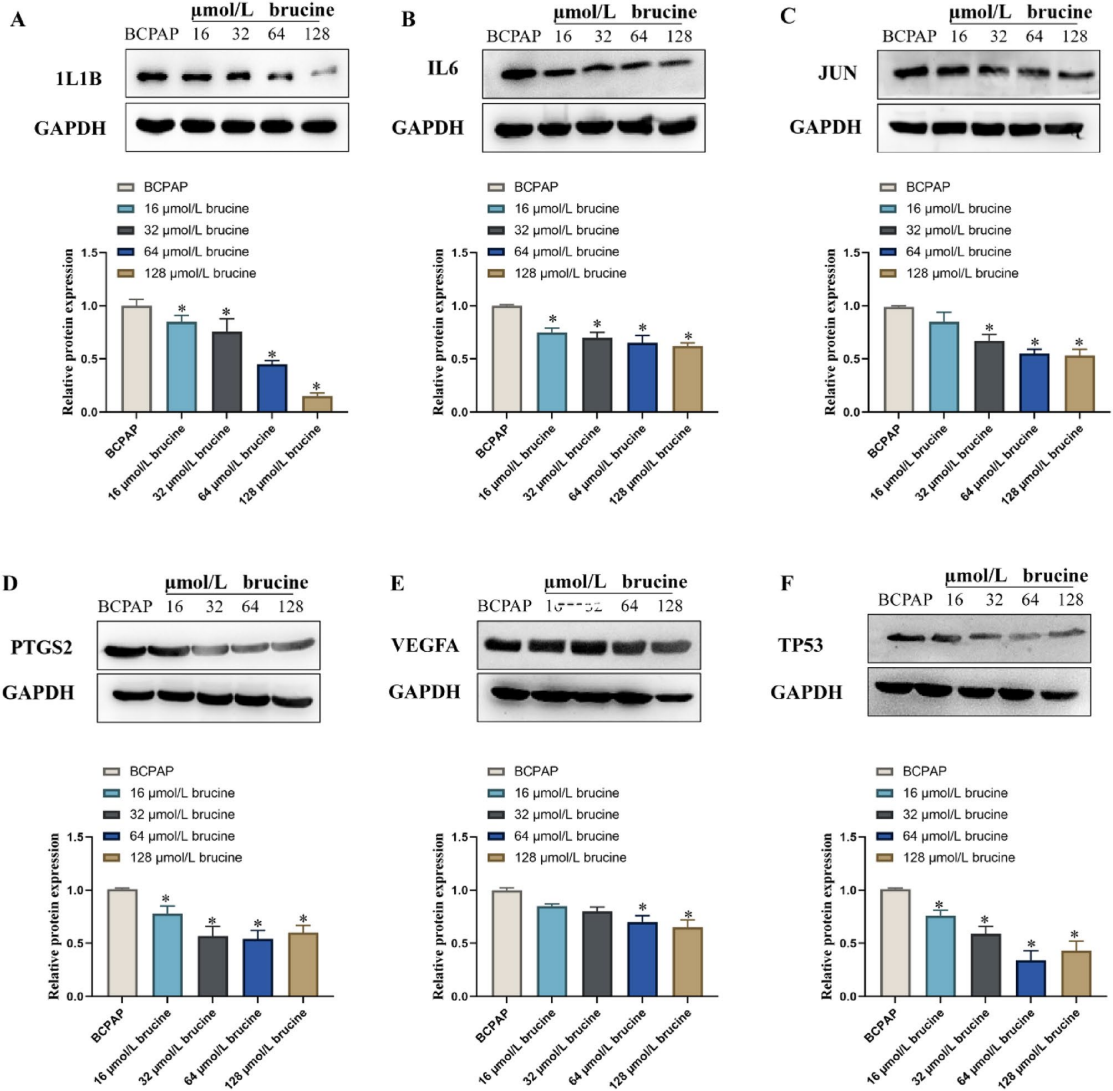
Effect of brucine on the expression of PTC-related proteins. (**A**) 1L1B, (**B**) IL6, (**C**) JUN, (**D**) PTGS2, (**E**) VEGFA, (**F**) TP53. *P < 0.05 was considered as statistically significant difference vs BCPAP cell.

In order to explore the mechanism of *SS* on anti-PTC, the protein expression levels of IL6, VEGFA, JUN, TP53, 1L1B and PTGS2 were detected in this study (Fig. 11). Compared with the control group, brucine at concentrations of 16, 32, 64 and 128 µmol/L significantly decreased the expression of 1L1B protein (Fig. 11A), IL6 protein (Fig. 11B), PTGS2 protein (Fig. 11D) and TP53 protein (Fig. 11E) (P < 0.05). At the same time, brucine with concentrations of 32, 64, and 128 µmol/L could significantly reduce the expression of JUN protein (P < 0.05) (Fig. 11C). Brucine with a concentration of 64 and 128 µmol/L could significantly reduce the expression of VEGFA protein (P < 0.05) (Fig. 11F).

#### Effect of brucine on the expression of apoptotic proteins

**Figure 12:**
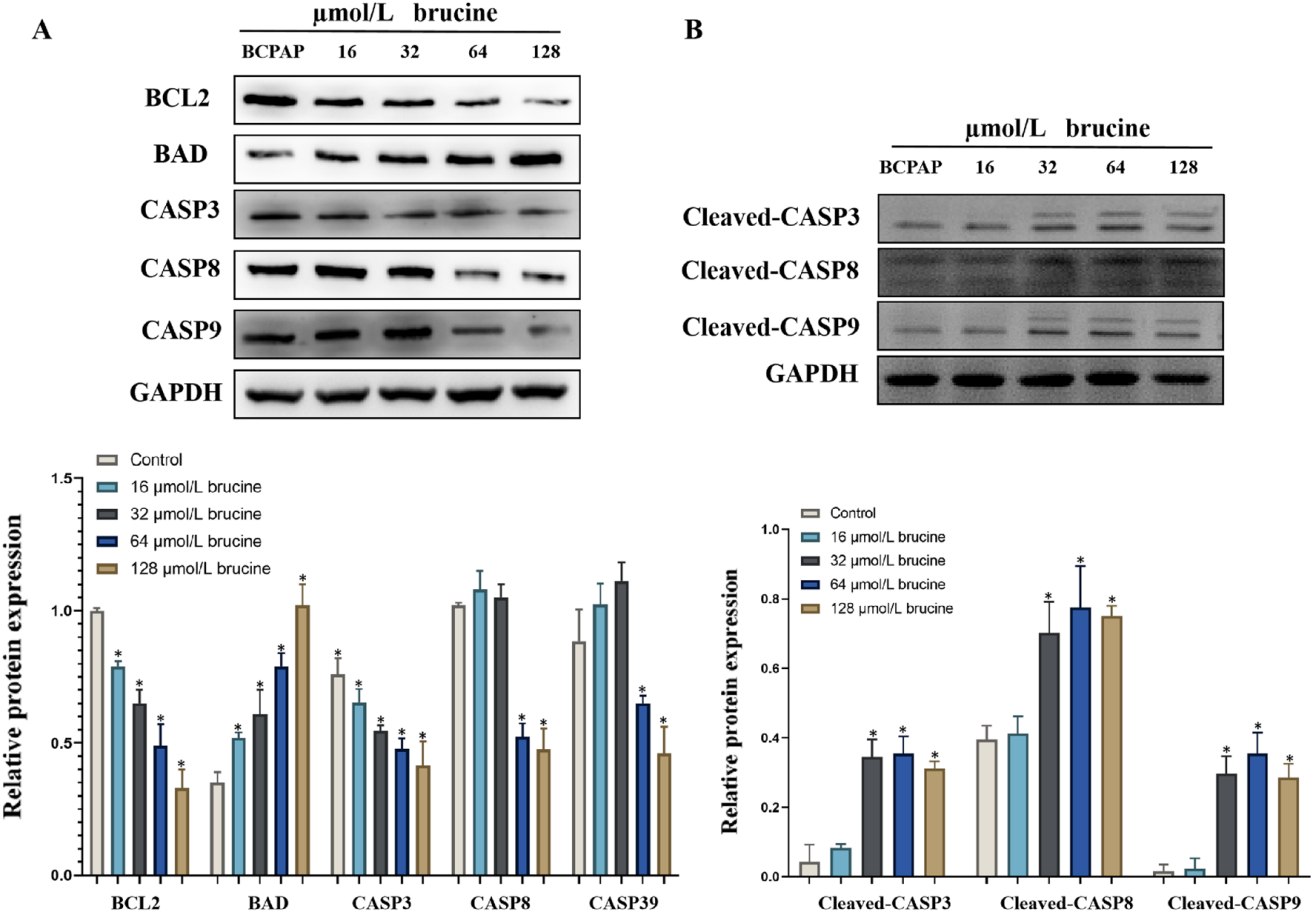
Effect of brucine on the expression of (**A**) apoptosis related proteins and (**B**) cleaved caspase proteins. *P < 0.05 was considered as statistically significant difference vs BCPAP cell.

For further investigated the molecular mechanism of brucine on anti-PTC, the cancer related apoptotic proteins were measured. The study results showed that the expressions of BCL2, CASP3, CASP8, CASP9 proteins were significantly increased while BAD, cleaved-CASP3, cleaved-CASP8, and cleaved-CASP9 proteins were significantly decreased which administrated with varies concentration (16, 32, 64, 128 µmol/L) of brucine (Fig. 12A,B).

## Discussion

In the present study, the active components of brucine, isobrucine, stigmasterol, and (+)-catechin from *SS* were predicted by network pharmacological method. It was reported that brucine exhibits a broad spectrum of anti-tumor effects. Brucine may inhibit cell migration, cell invasion and angiogenesis mimicry formation of human triple negative breast cancer cell line MDA-MB-231^26^. Previous study showed that brucine regulates Wnt/β-catechin signal pathway that can inhibit the growth and migration of colorectal cancer cell LoVo^27^, effectively inhibit the adhesion of liver cancer cells, and prevent the movement and invasion of HepG2 cells^28^. It was also found that brucine may significantly inhibit human leukemia K562 cell line and HL-60 cells, and induce apoptosis^29^.

In addition, isobrucine, stigmasterol, (+)-catechin the three potential pharmacological components may also play a synergistic anti-tumor effect, which deserves further experimental study. It was reported that isobrucine exhibits most potent cytotoxicity to tumor cell lines of MCF-7^30^. It was also revealed that isobrucine may make a contribution to the anti-proliferation on HepG2 cells, although they are present in *SS* with small quantities^31^. Previous research found that isobrucine showed the potential anti-cancer activities of tumor cell lines of K562, HeLa and HEP-2^32^. Recent studies on stigmasterol rich plant extracts have shown that they have significant anticancer effects on various tumor cell lines by inhibiting cell cycle progression and inhibiting cell growth by regulating cell proliferation. Especially in skin cancer^33^, gastric cancer^34^, lung cancer^35^, and liver cancer^36^, stigmasterol plays a prominent role through different mechanisms, and its activity seems to be dose dependent. Catechin is natural phenolic compound which is a phytopharmaceutical with promising anticancer effects but poor bioavailability^37^. Catechin have shown effectiveness as anti-inflammatory and anti-cancer, mainly through its activity to alter the pathway by NF-ΚB, Nrf-2, and MAPKs^38^. Compared with chemotherapy drugs, these catechin nanoparticles have relatively low systemic toxicity and are promising drugs with low side effects for cancer treatment. The anticancer activity of catechins has been demonstrated in various in vitro and in vivo cancer models with different potential molecules^39,40^. Taken together, above 4 active compounds of *SS* showed relatively strong broad-spectrum anti-cancer activity.

Through network pharmacological research, 6 key targets including IL6, VEGFA, JUN, TP53, 1L1B and PTGS2 were screened as important anti-PTC targets of *SS*. Through the construction of *SS* “drug-component-disease” target network and target gene PPI network, it was revealed that the target sites coordinated the process of proliferation, apoptosis, differentiation and metabolism to inhibit tumor metastasis through interaction. It has been demonstrated that the IL6 blockade potentiates the anti-tumor effects of γ-secretase inhibitors in Notch3-expressing can be abrogated by the IL6R blocking antibody tocilizumab in breast cancer^41^. It was also suggested that mucoepidermoid carcinoma patients might benefit from combination therapy with an inhibitor of IL-6R signaling and chemotherapeutic agent such as paclitaxel^42^. In previous study, it was revealed that IL-6 exhibits a significant role in thyroid cancer progression and targeting IL-6 signalling may be helpful in clinical management of thyroid carcinoma (TC) patients with more aggressive tumour characteristics^43^. It was showed that the expression of CD30L/CD30 is accompanied by the expression of IL-6/IL-6R signal. It may be of clinical significance to evaluate the expression of IL-6 protein in PTC and MTC, because of the expression level is related to tumor invasiveness^44^.

VEGFA protein has the biological activity of inhibiting the formation of new blood vessels. When tumor cells appear, its expression level will generally rise greatly. Some studies have shown that VEGFA is related to tumor infectivity and tumor susceptibility^45,46^. Previous research found that VEGFA gene and its protein product is widely expressed and increased in PTC and colloid goiter^45^. In addition, it was revealed that the molecular status of VEGFA may play a significant role in the progression of PTC^46^. JUN protein may be used as a specific diagnostic biomarker/therapeutic molecular target of PTC^47^. It was reported that the c-Jun was associated with the presence of extra-thyroid invasion and degree of tumor infiltration, while the T allele and acetylated c-Jun also correlated with tumor stage^48^. P53 gene is a kind of tumor suppressor gene. It is a negative regulator in cell growth cycle, and is related to cell cycle regulation, DNA repair, cell differentiation, apoptosis and other important biological functions. One of the most powerful obstacles to cancer is the normal function of p53. As a tumor suppressor gene, TP53 gene is a ubiquitous mutant gene, which may be involved in the formation and development of thyroid cancer^49^. Previous studies indicated that, the prevalence of homozygous Arg TP53 genotype in adult patients with radiation related PTC is significantly reduced compared with sporadic PTC cases and the general population, which suggests that other TP53 allele combinations may lead to the risk of papillary thyroid cancer in individuals with late childhood exposure^50^. IL1B is a key mediator for PTC to increase the release of immune regulatory factor prostaglandin E2 and tumor necrosis factor induced gene 6 protein and the expression level of various chemokine genes^51^. It was reported that IL1B polymorphism is risk factor for thyroid carcinoma in a Chinese Han population^52^. In previous study, it was found that the mRNA levels of PTGS2 encoding prostaglandin-endoperoxide synthase 2 increased in PTC, and an increased consumption of arachidonic acid was observed, which forms the oncogenic lipid in PTC^53^. It has been demonstrated that PTGS2 gene is detected in a large part of human thyroid cancer, which highlights the possibility of inhibiting tumor growth through COX-2 inhibition^54^. In the present study, we revealed that the expressions of IL6, VEGFA, JUN, TP53, 1L1B and PTGS2 were decreased by regulated by increase of the dose of brucine, isobrucine, stigmasterol, and (+)-catechin respectively.

Apoptosis refers to the programmed death of the body under the macro regulation of genes to maintain the stability of the internal environment of cells^55^. Previous research revealed that the pathogenesis of cancer is closely related to cell apoptosis^56^. Caspase-3 protein is one of the most important apoptotic executors among the caspase family and a major effector factor in the process of cell apoptosis. Its activation marks the irreversible stage of apoptosis^57^. Caspase-8 protein plays an important role in the development and progression of cancer, activating various structural and regulatory proteins involved in caspase-3 division^58^. These proteins are crucial for cell survival and maintenance, mediating and amplifying cascade reactions during apoptosis. It was reported that in the mitochondrial apoptosis pathway, when it receives a signal that is activated, it will cause cyto-c to enter the cytoplasm, bind with apaf-1 to form an apoptotic body, and induce caspase-9 activation. Finally, caspase-9 will transmit apoptosis information to the apoptotic executing protein caspase-3, triggering an apoptosis response^59^. Previous studies have shown that the bad protein is expressed in various cells and can participate in the entire process of cell apoptosis through cell signal transduction pathways and interactions with members of the caspase family^60^. In addition, as an anti-apoptotic factor, the physiological function of bcl-2 is to suppress cell apoptosis and prolong cell life. Related research shows that bcl-2 overexpression in cells can lead to changes in the nuclear redox balance, which plays a protective role by causing the accumulation of glutathione in the nucleus and reducing the activity of caspase^61^. In the present study, we further investigated the protein expression levels of BAD, BCL2, CASP3, CASP8, and CASP9, respectively. The results showed that brucine may significantly reduced the protein expression levels of BCL2, CASP3, CASP8, CASP9, and significantly increased the protein expression level of BAD. Furthermore, it was reported that cleaved caspase is the activated form of caspase which usually exists in cells undergoing apoptosis^62^. Therefore, the cleaved CASP3, cleaved CASP8, and cleaved CASP9 were detected respectively. The results revealed that brucine may increase the protein expression levels of BAD, cleaved-CASP3, cleaved-CASP8, and cleaved-CASP9 in BCPAP cells.

Further analysis on GO biological process enrichment and KEGG signal pathway of *SS* anti-tumor related targets showed that multiple signal pathways were related to anti-tumor, including IL-17 signaling pathway, endocrine resistance pathway, breast cancer, prostate cancer, etc. Through comparison, it was found that the most important pathways related to anti PTC mainly including TNF signaling pathway and MAPK signaling pathway. Previous researches showed that the representative endocrine resistance^63^, TNF signaling pathway^64^ and MAPK signaling pathway^65^ play an important role in the intracellular signal transduction pathway at all stages of tumor genesis and development, not only mediating cell activity through their own signal transduction process, but also jointly mediating complex biological activities in cells. The biological processes involved in the anti-PTC target of *SS* contain response to xenobiotic stimulus, mononuclear cell differentiation, positive regulation of transcription from RNA polymerase II promoter involved in cellular response to chemical stimulus, regulation of transcription from RNA polymerase II promoter in response to stress etc. Through the GO biological process enrichment analysis and KEGG signal pathway analysis of the anti-PTC related targets of *SS*, it has demonstrated that *SS* exerts its anti-tumor effect through multiple targets and multiple pathways.

Molecular docking is a method of drug design based on the characteristics of receptors and the interaction between receptors and drug molecules. In recent years, molecular docking has become an important technology in the field of computer-aided drug research^66,67^. In the present study, it has been demonstrated that the 4 active components of *SS*, isobrucine, stigmasterol, (+)-catechin and brucine, could bind to the 6 core protein targets in varying degrees. Experimental verification mainly concluding CCK8 test and Western blot test. CCK8 test results showed that brucine could significantly reduce the activity of BCPAP cells compared with isobrucine, stigmasterol, (+)-catechin. Western blot result showed that brucine could reduce the protein expression levels of IL-6, VEGFA, JUN, TP53, 1L1B, PTGS2, BCL2, CASP3, CASP8, CASP9 while increase the protein expression levels of BAD, cleaved-CASP3, cleaved-CASP8, and cleaved-CASP9 in BCPAP cells as the increase of dose, respectively.

## Conclusion

In the present study, the molecular mechanism effect of *SS* on anti-PTC was studied by screening the active components of *SS* and predicting the anti-tumor target, constructing the “component-disease-target” network diagram, constructing the PPI network, and conducting GO, KEGG analysis and other network pharmacological methods on the target. It was successfully predicted the main active components of *SS* anti-PTC effect, anti-PTC target and its related signal pathways and biological processes. It reflects the complex mechanism of multi-component and multi-target effects of TCM on diseases. To our knowledge, this is the first application of in silico molecular docking to identify a small molecular and compounds from *SS* in treatment of PTC-related cells. At the same time, the manuscript also studied the expression of active ingredient brucine in *SS* on the target protein through experimental verification methods, proving that the active ingredient may act on PTC through related pathways, which provides a research basis for *SS* anti-PTC.

### Supplementary Information

Supplementary Information.

## Data Availability

All data included or relevant to the study are available upon request by contact with the corresponding author. In addition, the datasets which supposing the present study are available in public database from TCMSP (https://tcmsp-e.com/tcmspsearch.php?qr=Strychni%20Semen&qsr=herb_en_name&token=26ae8d732e18e08a835c7891b4563d1a), SwissTarget Prediction (http://www.swisstargetprediction.ch/result.php?job=1900714187&organism=Homo_sapiens), SEA (https://sea.bkslab.org/jobs/search_b47fb92d-892a-404c-a9a6-7df735649bd7), Genecards (https://www.genecards.org/), UniProt (http://www.uniprot.org/), String (https://cn.string-db.org/), Metascape (http://metascape.org/), DAVID (https://david-d.ncifcrf.gov/home.jsp), PubChem (https://pubchem.ncbi.nlm.nih.gov/), GEPIA (http://gepia.cancer-pku.cn/), TCGA (http://portal.gdc.cancer.gov/), GTEx (http://gtexportal.org/home/), GEPIA2 (http://gepia.cancer-pku.cn/), and PDB (https://www.rcsb.org/) respectively.
