# Supplementary material for: Systematic pharmacology-based strategy to explore the mechanism of Semen Strychni for treatment of papillary thyroid carcinoma
Source: Sci Rep. 2023 Oct 28;13:18492. doi: 10.1038/s41598-023-45741-9 (PMC10613225; doi:10.1038/s41598-023-45741-9)
Supplement: Supplementary file 1 — Supplementary Information. [file 41598_2023_45741_MOESM1_ESM.docx]

**Systematic pharmacology-based strategy to explore the mechanism of *Semen Strychni* for treatment of papillary thyroid carcinoma**

**Jingxin Mao^1,2#^, Lijing Tang^1,3^, Ling Fang^1,3^, Cheng Tian^1,3^, Zhaojing Zhu^1,3^, Yan Li^1,3*^**

^1^ Chongqing Medical and Pharmaceutical College, Chongqing 400030, China.

^2^ College of Pharmaceutical Sciences, Southwest University, Chongqing 400715, China.

^3^ Chongqing Key Laboratory of High Active Traditional Chinese Drug Delivery System, Chongqing 400030, China.

^*^**Corresponding author**: Yan Li, associate professor.

Chongqing Medical and Pharmaceutical College, No. 82, Middle University Town Road, Shapingba District, Chongqing 400030, China.

**Email**: liyan77777777@126.com or 10864@cqmpc.edu.cn

Tel:+86-18580137767

**Running title: Mechanism of *SS* on PTC**

**List of supporting information**

S1 Chemical property of brucine.

S2. Purity analysis of brucine.

Figure S1. Purity analysis of brucine.

Figure S2. The structure of (A) brucine, (B) isobrucine, (C) (+)-catechin, (D) stigmasterol respectively.

Figure S3. The images of the original western blots of (A) 1L1B and (B) GAPDH.

Figure S4. The images of the original western blots of (A) IL6 and (B) GAPDH.

Figure S5. The images of the original western blots of (A) JUN and (B) GAPDH.

Figure S6. The images of the original western blots of (A) PTGS2 and (B) GAPDH.

Figure S7. The images of the original western blots of (A) VEGFA and (B) GAPDH.

Figure S8. The images of the original western blots of (A) TP53 and (B) GAPDH.

Figure S9. The images of the original western blots of BAD.

Figure S10. The images of the original western blots of BCL2.

Figure S11. The images of the original western blots of CASP3.

Figure S12. The images of the original western blots of CASP8.

Figure S13. The images of the original western blots of CASP9.

Figure S14. The images of the original western blots of GAPDH.

Figure S15. The images of the original western blots of cleaved CASP3.

Figure S16. The images of the original western blots of cleaved CASP8.

Figure S17. The images of the original western blots of cleaved CASP9.

Figure S18. The images of the original western blots of GAPDH.

S1 Chemical property of brucine.

**ACS of brucine: 357-57-3**

Brucine, colorless crystal, odorless, extremely bitter in taste, slightly soluble in water, ethanol, acetone, and insoluble in ether. Molecular formula: C_23_H_26_N_2_O_4_, molecular weight: 394.5, melting point: 180℃, colorless needle crystal.

S2 Purity analysis of brucine standard.

Shimadzu LC-20A high performance liquid chromatography with dual solvent pump high-pressure gradient system, SPD-20A photodiode array detector, and an autosampler were used for the first-dimension separation.

Brucine standard was accurately weighed and dissolved in methanol with the final concentrations at was 1.0 mg/mL were prepared and stored at 4 ℃ before analysis. The brucine standard was filtered with 0.5 μm organic membrane to prepare for HPLC analysis. Chromatographic elution was conducted with binary mobile phase gradient consisting of ethanol solvent. Initial gradient conditions were set to 5% ethanol at the ﬂow rate of 1.0 mL/min, gradient increasing to 100% ethanol over 60 min. The column temperature was maintained at 37 ℃ during the whole process. Analysis chromatographic column Shimadzu C18 (250mm × 4.6mm, 5 μm) was used and the detection wavelength is 254 nm. The volume of brucine standard injection is 20 μL. The retention time of brucine standard is 16 min.

Figure S1. Purity analysis of brucine standard.

A: The HPLC of brucine standard which was purchased from Sigma-Aldrich China Co., LLC. (Shanghai, China).


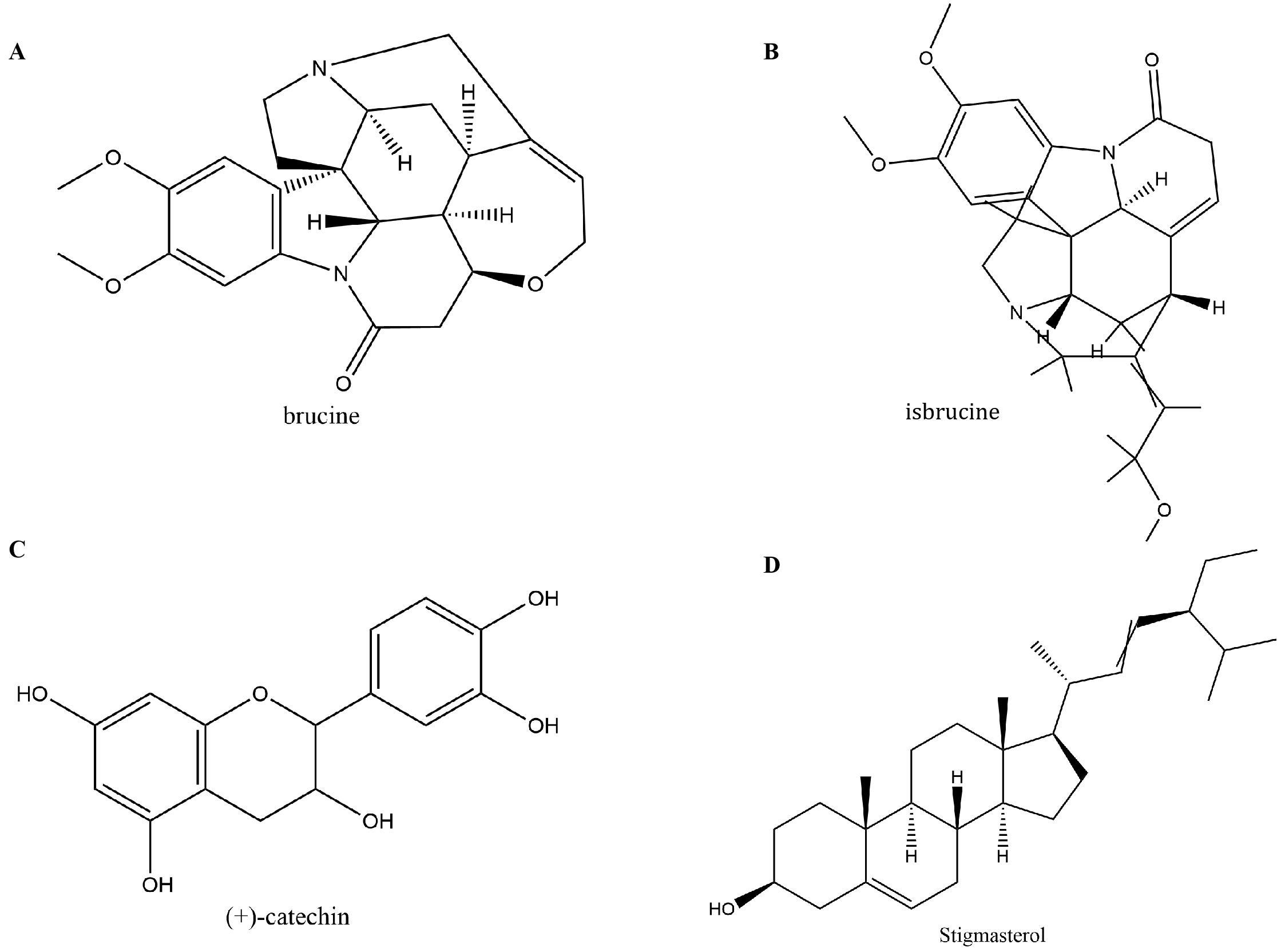


Figure S2. The structure of (A) brucine, (B) isobrucine, (C) (+)-catechin, (D) stigmasterol respectively.

1.
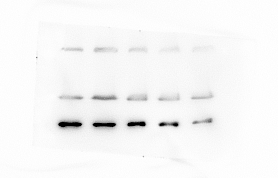

2.
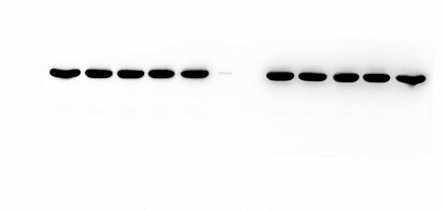


Figure S3. The images of the original western blots of (A) 1L1B and (B) GAPDH.

1.
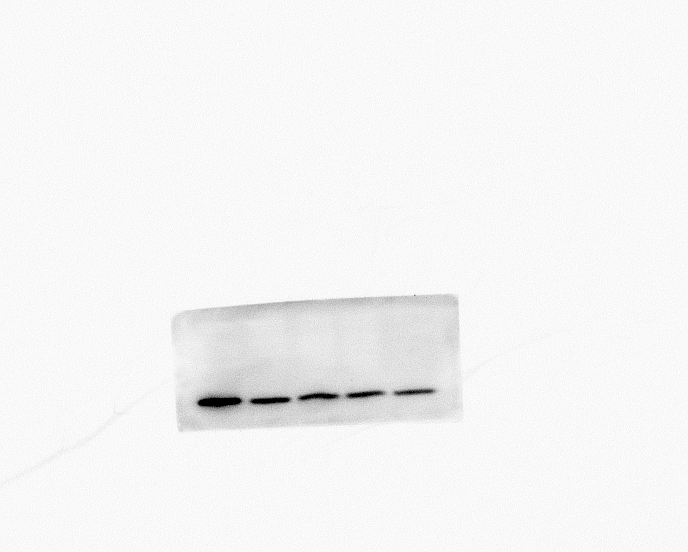


(B)
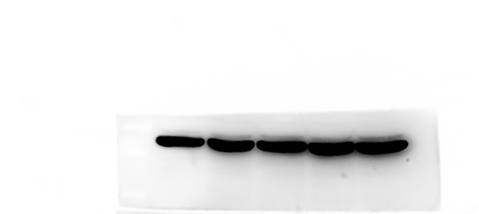


Figure S4. The images of the original western blots of (A) IL6 and (B) GAPDH.

1.
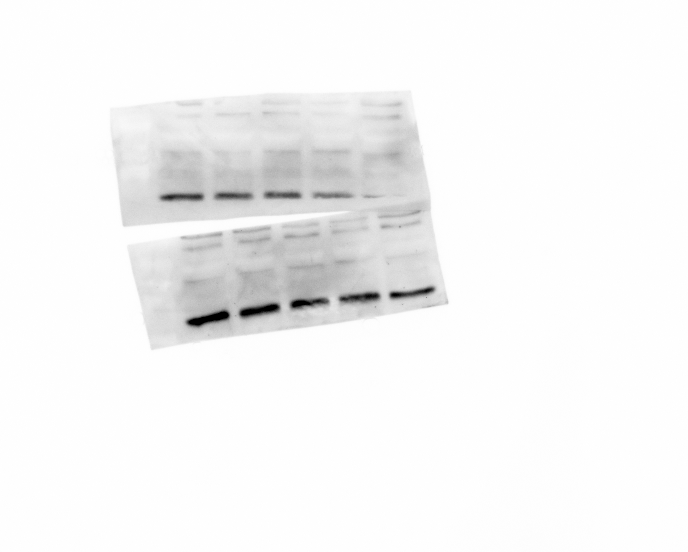

2.
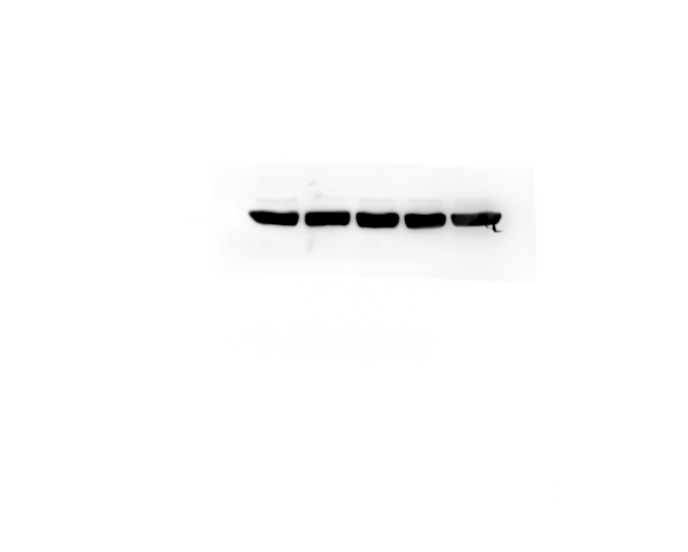


Figure S5. The images of the original western blots of (A) JUN and (B) GAPDH.

1.
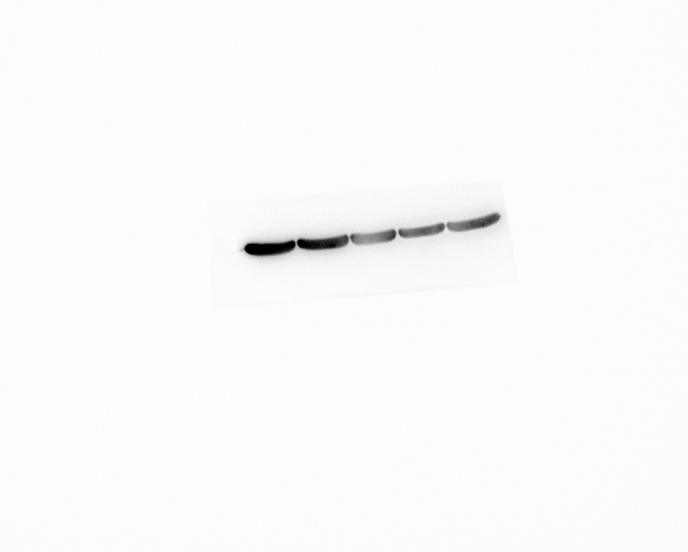

2.
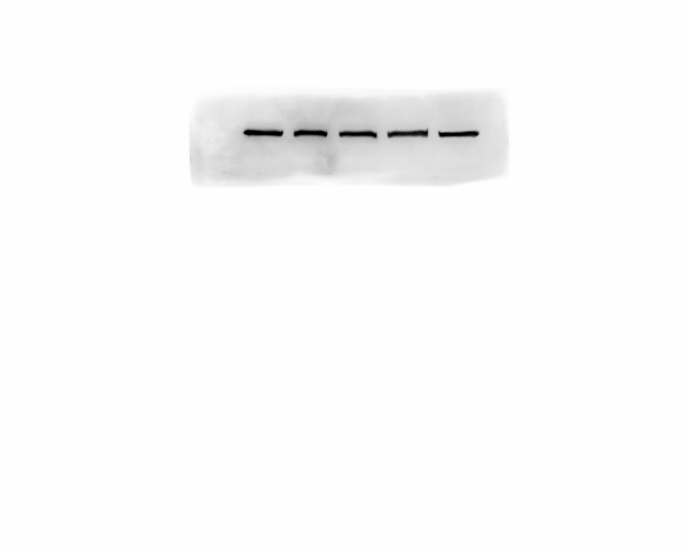


Figure S6. The images of the original western blots of (A) PTGS2 and (B) GAPDH.

1.
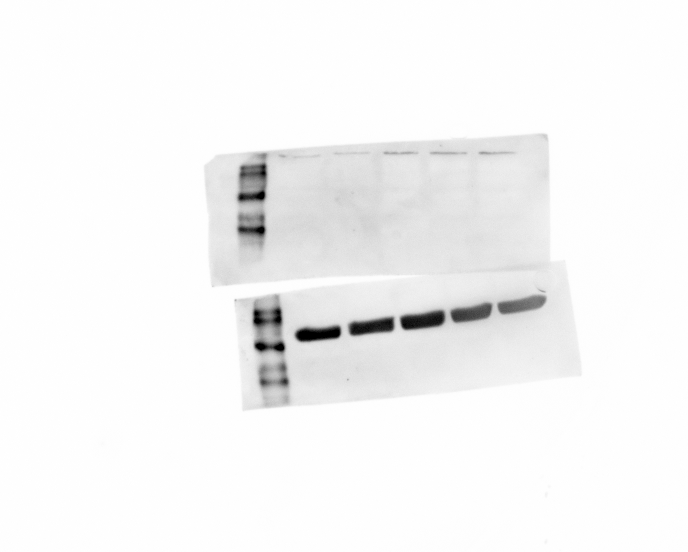

2.
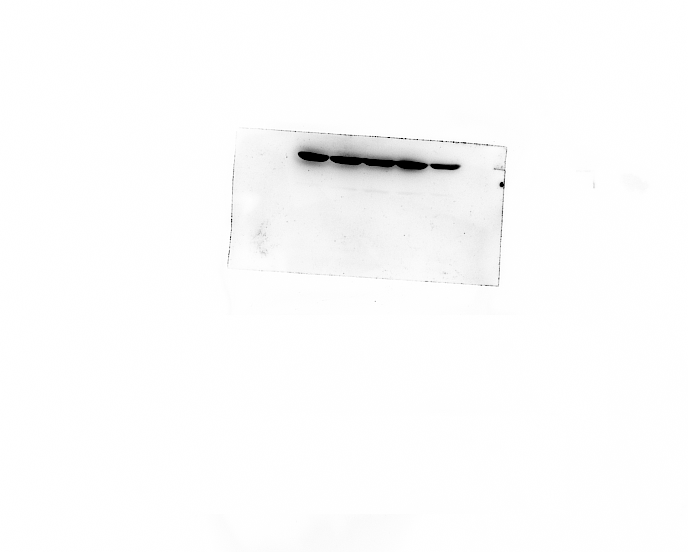


Figure S7. The images of the original western blots of (A) VEGFA and (B) GAPDH.

1.
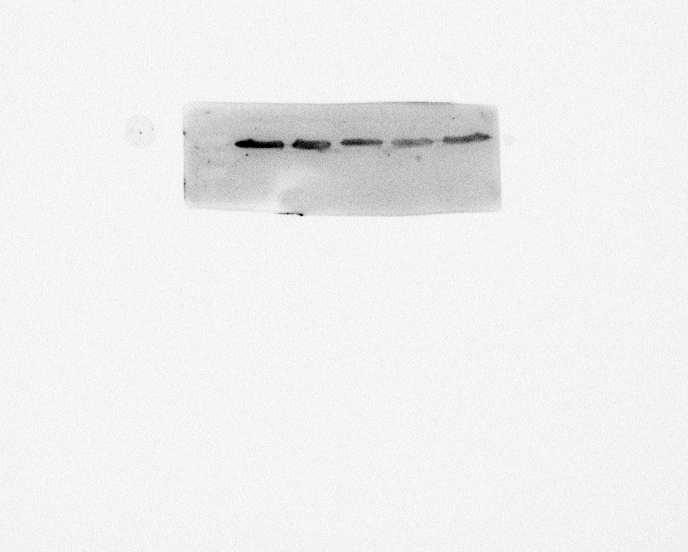

2.
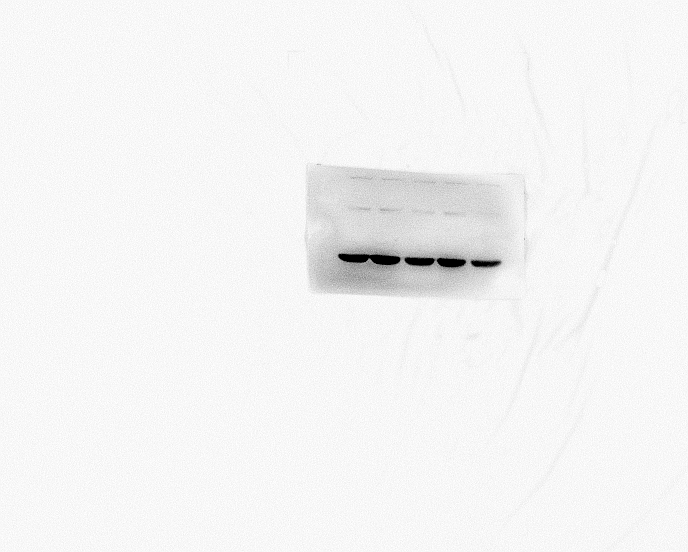


Figure S8. The images of the original western blots of (A) TP53 and (B) GAPDH.


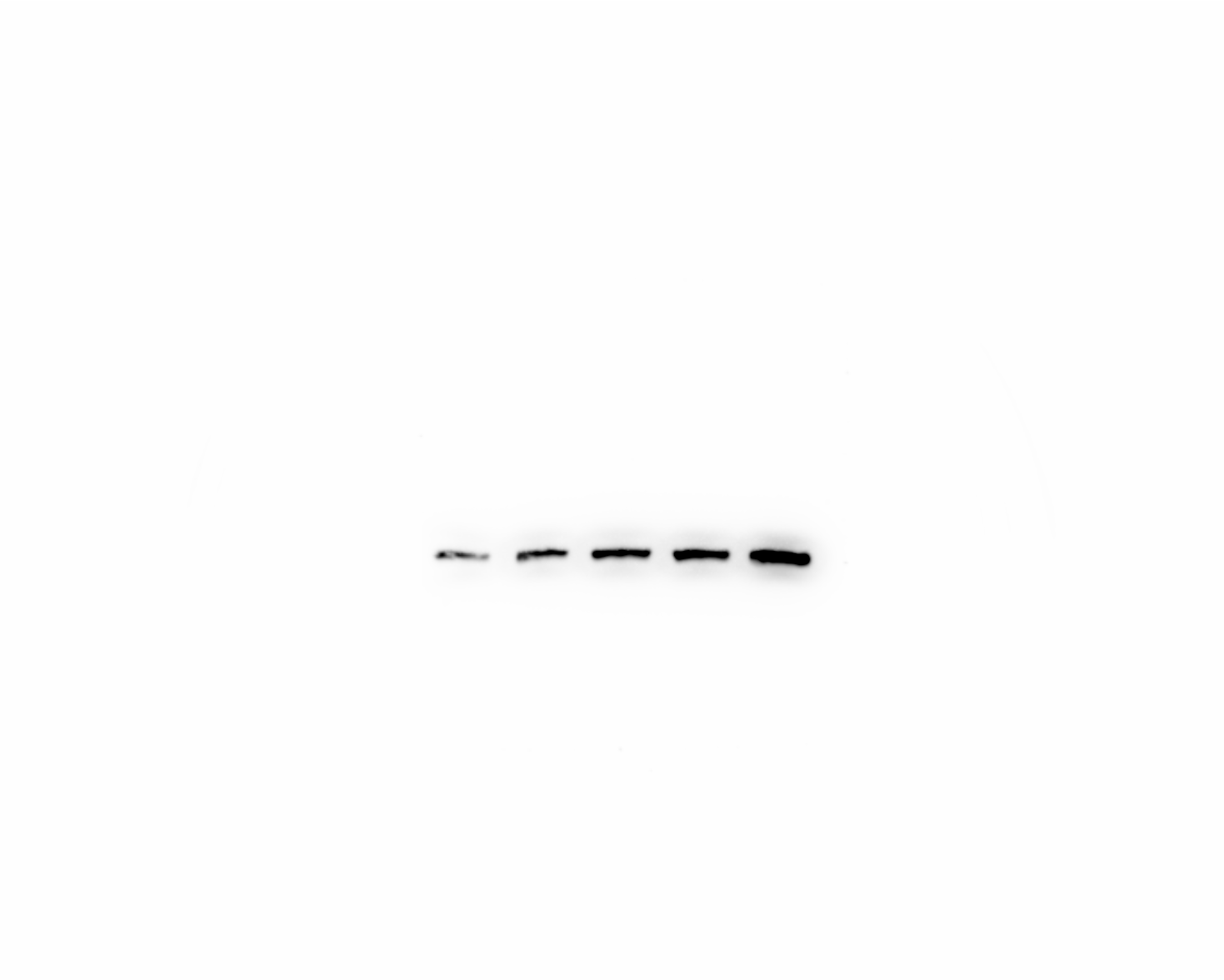


Figure S9. The images of the original western blots of BAD.


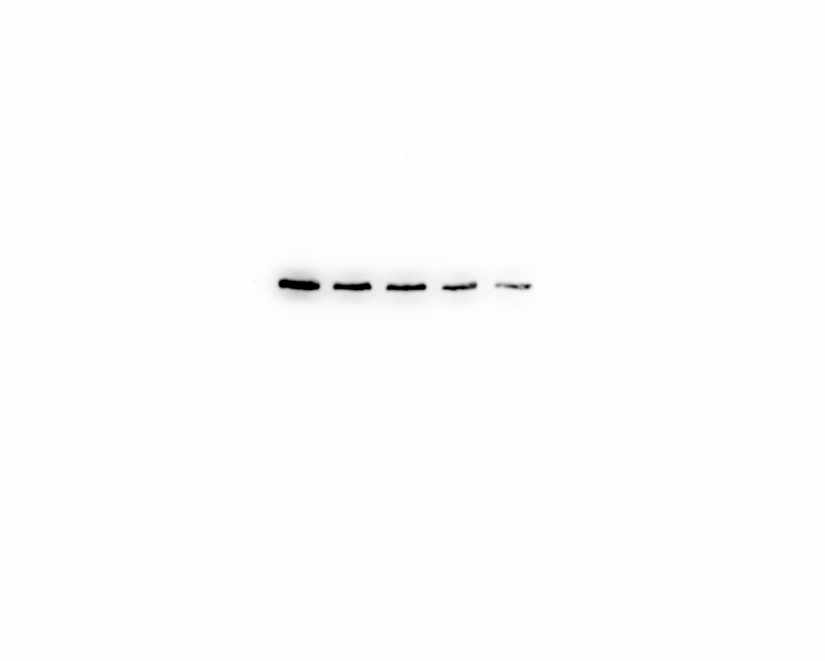


Figure S10. The images of the original western blots of BCL2.


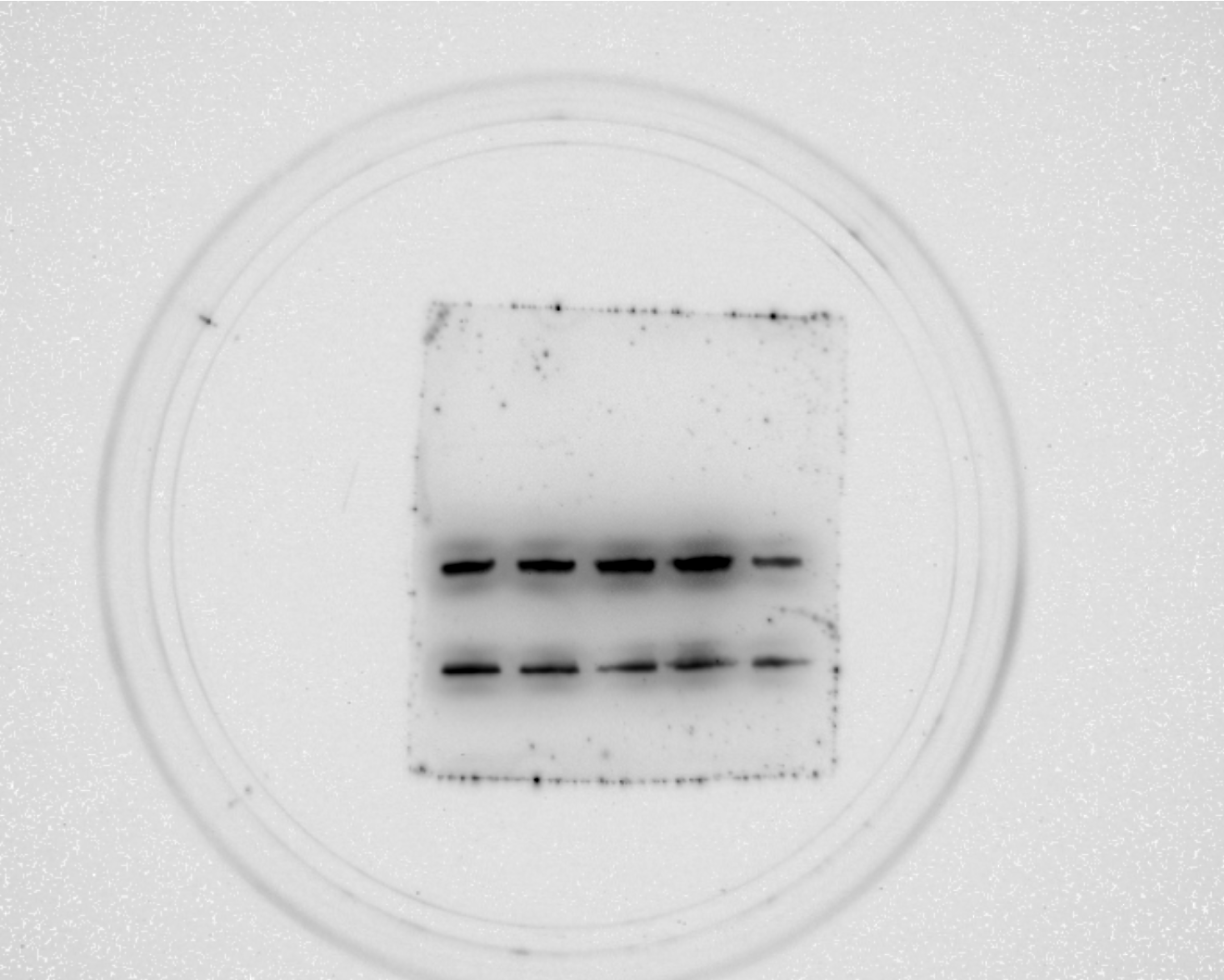


Figure S11. The images of the original western blots of CASP3.


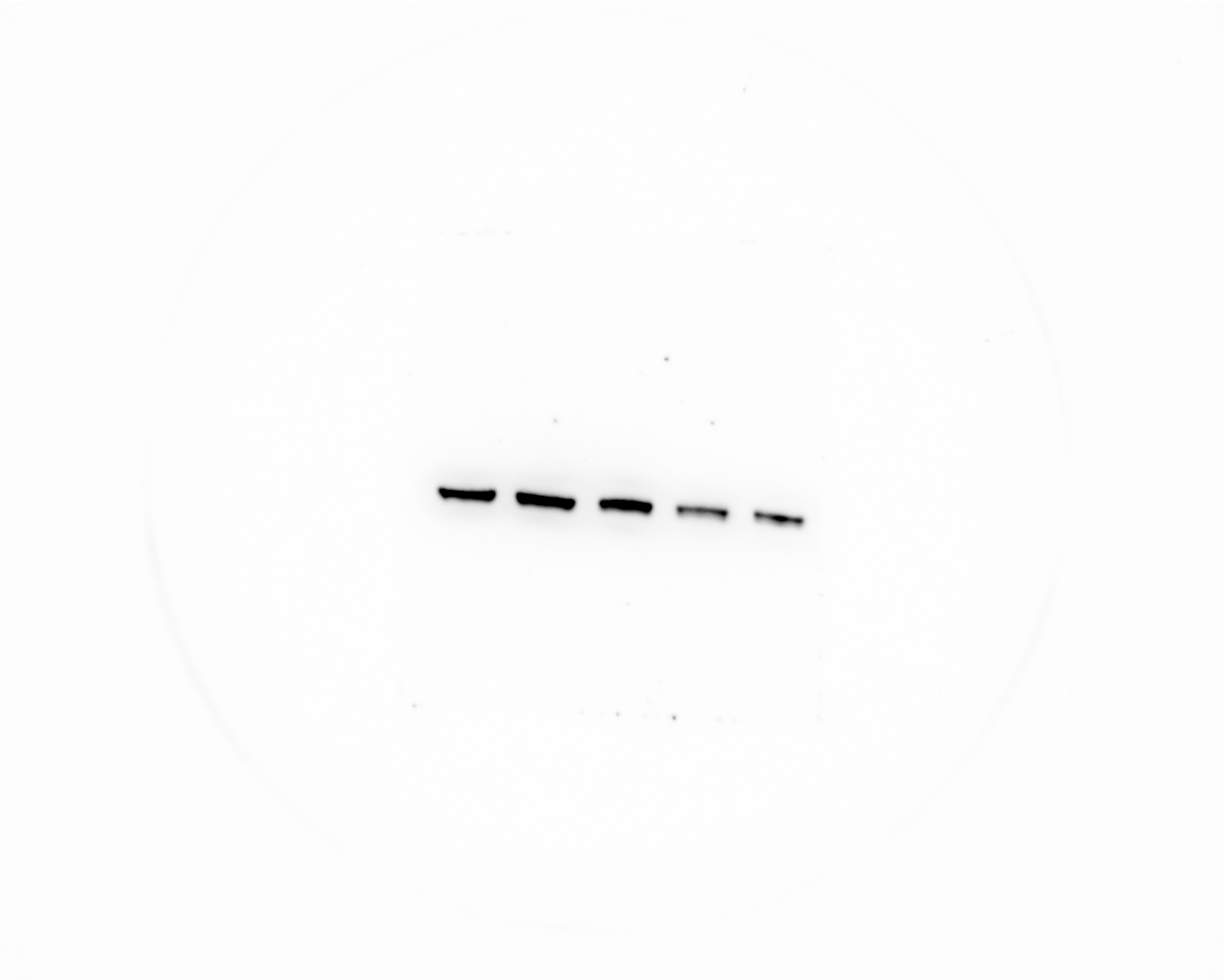


Figure S12. The images of the original western blots of CASP8.


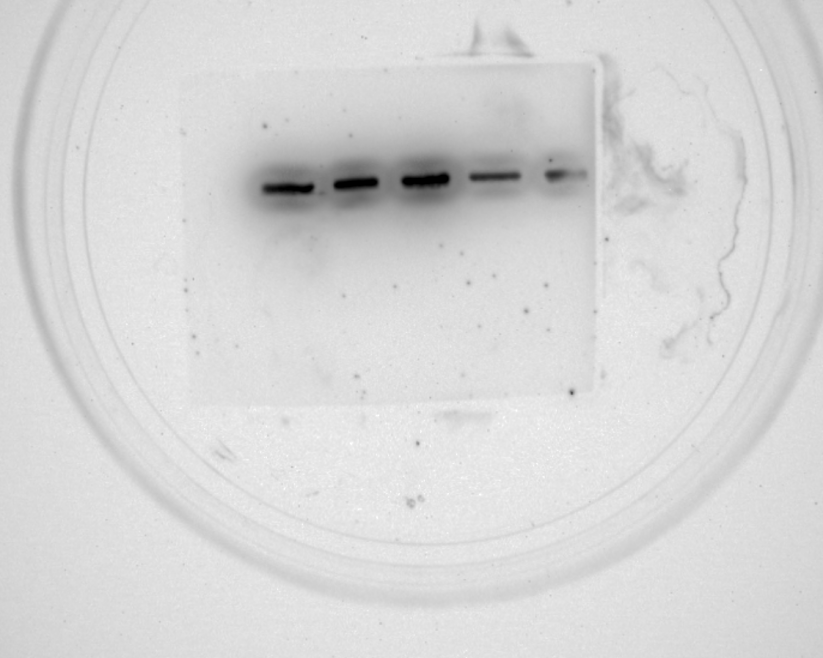


Figure S13. The images of the original western blots of CASP9.


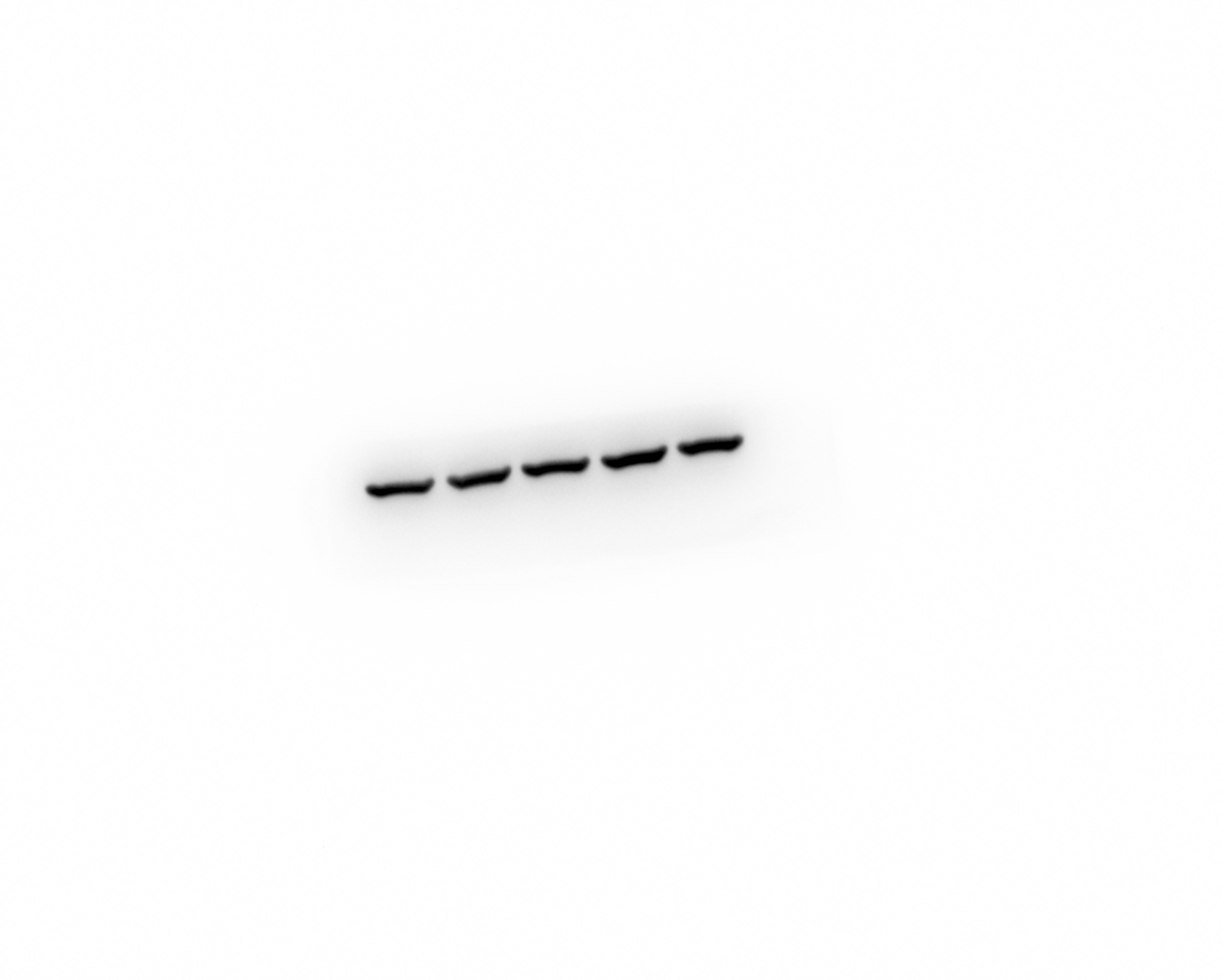


Figure S14. The images of the original western blots of GAPDH.


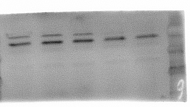


Figure S15. The images of the original western blots of cleaved CASP3.


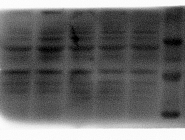


Figure S16. The images of the original western blots of cleaved CASP8.


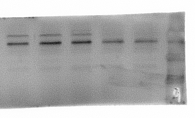


Figure S17. The images of the original western blots of cleaved CASP9.


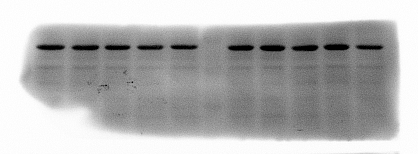


Figure S18. The images of the original western blots of GAPDH.
